# Process‐Informed Neural Networks: A Hybrid Modelling Approach to Improve Predictive Performance and Inference of Neural Networks in Ecology and Beyond

**DOI:** 10.1111/ele.70012

**Published:** 2024-12-03

**Authors:** Marieke Wesselkamp, Niklas Moser, Maria Kalweit, Joschka Boedecker, Carsten F. Dormann

**Affiliations:** ^1^ Biometry and Environmental System Analysis University of Freiburg Freiburg im Breisgau Germany; ^2^ Biological and Environmental Science University of Jyväskylä Jyväskylä Finland; ^3^ Department of Computer Science University of Freiburg Freiburg im Breisgau Germany; ^4^ Cluster of Excellence BrainLinks‐BrainTools Freiburg im Breisgau Germany

**Keywords:** deep learning, environmental prediction, explainable AI, inference, neural network, physics‐informed, prediction, process model, process‐informed, transferability

## Abstract

Despite deep learning being state of the art for data‐driven model predictions, its application in ecology is currently subject to two important constraints: (i) deep‐learning methods are powerful in data‐rich regimes, but in ecology data are typically sparse; and (ii) deep‐learning models are black‐box methods and inferring the processes they represent are non‐trivial to elicit. Process‐based (= mechanistic) models are not constrained by data sparsity or unclear processes and are thus important for building up our ecological knowledge and transfer to applications. In this work, we combine process‐based models and neural networks into process‐informed neural networks (PINNs), which incorporate the process knowledge directly into the neural network structure. In a systematic evaluation of spatial and temporal prediction tasks for C‐fluxes in temperate forests, we show the ability of five different types of PINNs (i) to outperform process‐based models and neural networks, especially in data‐sparse regimes with high‐transfer task and (ii) to inform on mis‐ or undetected processes.

## Graphical abstract

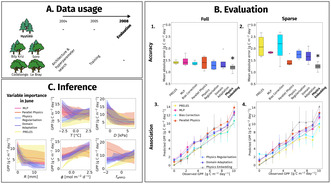

## 1 Introduction

Ecology seeks to understand the causes of species abundances and distributions, based on the interactions with the environment and each other (Haeckel 1866; Krebs 2002). Identifying the mechanisms is challenging, given the diversity of phenomena, the multitude of environmental drivers and the variability of organisms involved (Urban et al. 2017). Ecological research has thus always reached across the entire spectrum of causal to correlative approaches and sought ways to describe ecological patterns from a statistical‐correlative angle, as well as from a mechanistic one (Geary et al. 2020). In recent years, the predictive ability of ecological models has gained more prominence (Clark and Gelfand 2006; Boettiger 2022), both as a means to test our understanding under new settings (Houlahan et al. 2017; Getz et al. 2018) and as a service to policy (Schindler and Hilborn 2015). Indeed, Currie (Currie 2019) and Lewis et al. (Lewis et al. 2023) argue that progress in ecology comprises both aspects: getting the prediction right *and* deriving testable hypotheses from such correct statistical models.

Increasing amounts of data of ecological interest have forged a variety of big data activities across ecological disciplines such as macroecology (Wüest et al. 2020), ecosystem ecology (Pastorello et al. 2020) and movement ecology (Kays et al. 2022). Consequently, deep‐learning methods have become popular wherever data availability supports it (for a comprehensive review, see 15). Yet, there is often only little or abstract mechanistic insight into the processes yielding the data. Furthermore, in many ecological disciplines, data still remain comparatively sparse and incomplete. Most ecological and environmental systems are driven by processes that occur at several spatial and temporal scales and produce observable patterns at varying scales (Levin 1992). Rarely are these scales monitored and measured, resulting in a patchy representation of ecological processes.

After centuries of ecological research, there are several fields with substantial process understanding (reviewed in 17), such as fisheries (Jennings, Kaiser, and Reynolds 2009; Fogarty and Collie 2020), forest growth (Medlyn, Duursma, and Zeppel 2011; Forrester 2019), carbon fluxes (Friend et al. 2007; Sitch et al. 2008), ecohydrology (Guswa, Celia, and Rodriguez‐Iturbe 2002; Fatichi, Pappas, and Ivanov 2016), individual‐based models (Accolla et al. 2021; Malishev and Kramer‐Schadt 2021) and animal energetics (Kooijman 2010; Kearney, Domingos, and Nisbet 2015). Representing data by purely statistical means and deep learning does not leverage the opportunity of further improving this ecological understanding (Wikle and Hooten 2010). Machine and deep learning may be powerful tools for prediction given big data (Reichstein et al. 2019) but provide no or only limited improvements over traditional statistical methods for small to moderately sized datasets (Faraway and Augustin 2018; Bury et al. 2021). In a predictive setting, they may well be ‘right for the wrong reason’, that is, instead of the true causal drivers merely using correlated and hence spurious surrogates (McCoy, Pavlick, and Linzen 2019). Prone to learning frequent rather than specific pattern in the data, highly flexible deep‐learning methods are thus expected to have low transferability (Pichler and Hartig 2023a; McCoy, Pavlick, and Linzen 2019; Karlbauer et al. 2022), unless severely regularised (Belkin et al. 2019). Peeping into successful models using ‘explainable AI’ is a start (Ryo et al. 2021), but we believe we can do, and already have done, much better.

Deep learning has opened another route to joining correlative flexibility and process understanding (Reichstein et al. 2019) using what is sometimes called ‘process‐guided neural networks’ (or, in physics, ‘physics‐informed NN’: PINN (Raissi, Perdikaris, and Karniadakis 2019) or, in other fields, ‘knowledge‐guided machine learning’: KGML (Liu et al. 2022)), deep learning augments existing process description and attempts to improve on it (Liu et al. 2022; Ba, Zhao, and Kadambi 2019; Karpatne et al. 2018; Zhao et al. 2019; Karniadakis et al. 2021). The resulting mixture of process and correlative model builds on our current understanding of a system, yet it offers the flexibility to modify and enrich such system descriptions (Karniadakis et al. 2021). As a result, the improvements achieved by the statistical component can help identify what is missing in the model and suggest elements to be trialled in silico or experimentally.

This approach is not new and has been used even in ecology, typically in the form of partially specified models (Wood 2001) or as components of hierarchical models in Bayesian frameworks (Clark and Gelfand 2006; Wikle and Hooten 2010; Hefley, Hooten et al. 2017). New is, however, that deep learning frameworks such as PyTorch facilitate a richer array of PINN structures with physical information being integrated at different aspects of the algorithmic pipeline. These approaches may have the potential to reduce data requirements (Raissi, Perdikaris, and Karniadakis 2019), the cost of suitability for sparse data settings and uncertainty quantification compared with deep hierarchical models (Wikle and Hooten 2010; Hefley, Broms et al. 2017), unless Bayesian or Ensemble PINNs are used (Psaros et al. 2022; Zou, Meng, and Karniadakis 2023). To our knowledge, no systematic study has examined the use of PINNs in (ecosystem) ecology, comparing their on‐site predictive performance, generalisation beyond the training site or ability to reveal unknown processes.

**FIGURE 1:**
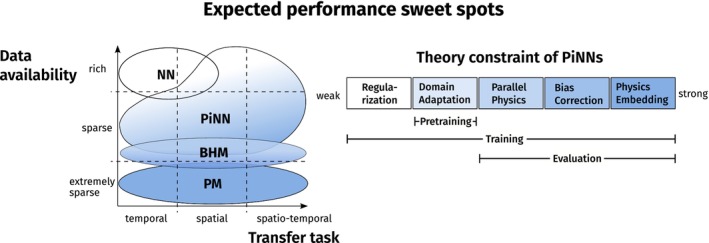
Schematic overview of expected performance sweet spots of neural networks (NN), process‐based models (PM), Bayesian hierarchical models (BHM) and physics‐informed neural networks (PINN). We differentiate between five PINNs with increasing theory constraint for which we expect performance sweet spots, especially in data‐sparse regimes and high‐transfer tasks, and highlight parts of the algorithmic pipeline in which the constraint applies.

When correlative models were touted as fitting data better and process‐based models (PMs) being superior in extrapolation (Kearney, Domingos, and Nisbet 2015; Mouquet et al. 2015), they often employed the gradient in data availability as argument: with lots of data, statistical models can effectively represent the information and make accurate predictions. At the other extreme, with only a handful of data points, correlative models are undercomplex, while process‐based models will outperform them (Figure 1) (Dormann et al. 2012). This gradient of data availability does not, in our view, call for a switch from one model type to another but supports a blended approach (Wikle and Hooten 2010).

In the following, we demonstrate from a deep‐learning perspective how predictive skill and theory development can be fruitfully combined, using PINNs with an ecological case study. First, we briefly review existing approaches of linking process and statistical models. Second, using the example of forest carbon fluxes, we evaluate the extrapolation skill of PINNs, compared to a purely process and a purely correlative reference, both in time and in space, using a full and a sparse dataset. We then explore what the correlative aspect of the PINNs can reveal about misspecifications in the PM, allowing us to generate testable hypotheses from this combined approach. We close with an outlook of current challenges in this field and arising options for alternative PINN architectures.

## 2 Methods Overview

The methodological extremes in the context of process‐informed neural networks (PINNs) are set by entirely data‐driven correlative models (regression, machine learning and deep learning) on one side and entirely ‘forward’ process‐based models (process‐based model: PM) on the other (Dormann et al. 2012). ‘Forward’ implies that no data were used to calibrate, fit or fine‐tune the model parameters to the observed data at hand, although during model development many such data were used, formally or informally, to derive the model's default parameterisation.

### 2.1 Fitted Process‐Based Models

One step away from the ‘forward’ PMs are fitted PMs, where the model parameters are optimised to describe the training data (Cabral, Valente, and Hartig 2017), routinely done with, for example, population dynamic models (Kéry and Royle 2020).

### 2.2 Partial Process‐Based Models

In partial PMs, the primary explanatory power is attributed to the PM. This has most clearly been described from a hierarchical Bayesian perspective, where the framework of ‘General quadratic non‐linearity’ accommodates the mechanistic and statistical aspects of spatio‐temporal problems in a shared formalisation (Wikle and Hooten 2010). The correlative elements are applied to the residuals, that is, the difference between observation y and process‐model expectation ${\hat{y}}_{\mathrm{PM}}$. Depending on whether the PM parameters are fitted alongside, approaches can be separated into ‘residual models’ (Ba, Zhao, and Kadambi 2019) and integrated models (Wood 2001; Schaub and Abadi 2010). Based on the former, we develop a ‘parallel physics’ neural network (NN) that optimises the loss ℒ function with PM predictions ${\hat{y}}_{\mathrm{PHY}}$, NN predictions ${\hat{y}}_{\mathrm{NN}}$ and observations y as

(1) $$ℒ={ℒ}_{\mathrm{MSE}}\left(y,{\hat{y}}_{\mathrm{NN}}+{\hat{y}}_{\mathrm{PHY}}\right).$$

This approach subtracts the static PM's prediction from the response or, when the response variable is non‐normal, uses it as an offset in the statistical model. In Box 1, we exemplify this by partially integrating a continuous Lotka–Volterra system with an NN.

### 2.3 Bias Correction

Even simpler is bias correction, where the output from the PM is used as predictor(s) in a regression‐type model. The idea here is that consistent differences (bias) in PM prediction ${\hat{y}}_{\mathrm{PHY}}$ can be calibrated with observed data y in a post‐processing manner (Rasp and Lerch 2018). The regression model takes as input the (potentially multidimensional) set of ${\hat{y}}_{\mathrm{PHY}}$ and learns to minimise the differences between estimates for ${\hat{y}}_{\mathrm{NN}}$ and y, simply given by the loss function

(2) $$ℒ={ℒ}_{\mathrm{MSE}}\left(y,{\hat{y}}_{\mathrm{NN}}\right).$$

Note that the dimensionalities of ${\hat{y}}_{\mathrm{PHY}}$ and ${\hat{y}}_{\mathrm{NN}}$ can differ. In our case studies, ${\hat{y}}_{\mathrm{PHY}}$ consists of three state variables, the gross primary productivity, the evapotranspiration and the soil water content ($\left\{P,E,\theta \right\}$, respectively), whereas in y and ${\hat{y}}_{\mathrm{NN}}$ we target only one state variable, namely P.

### 2.4 Physics Regularisation

**FIGURE 2:**
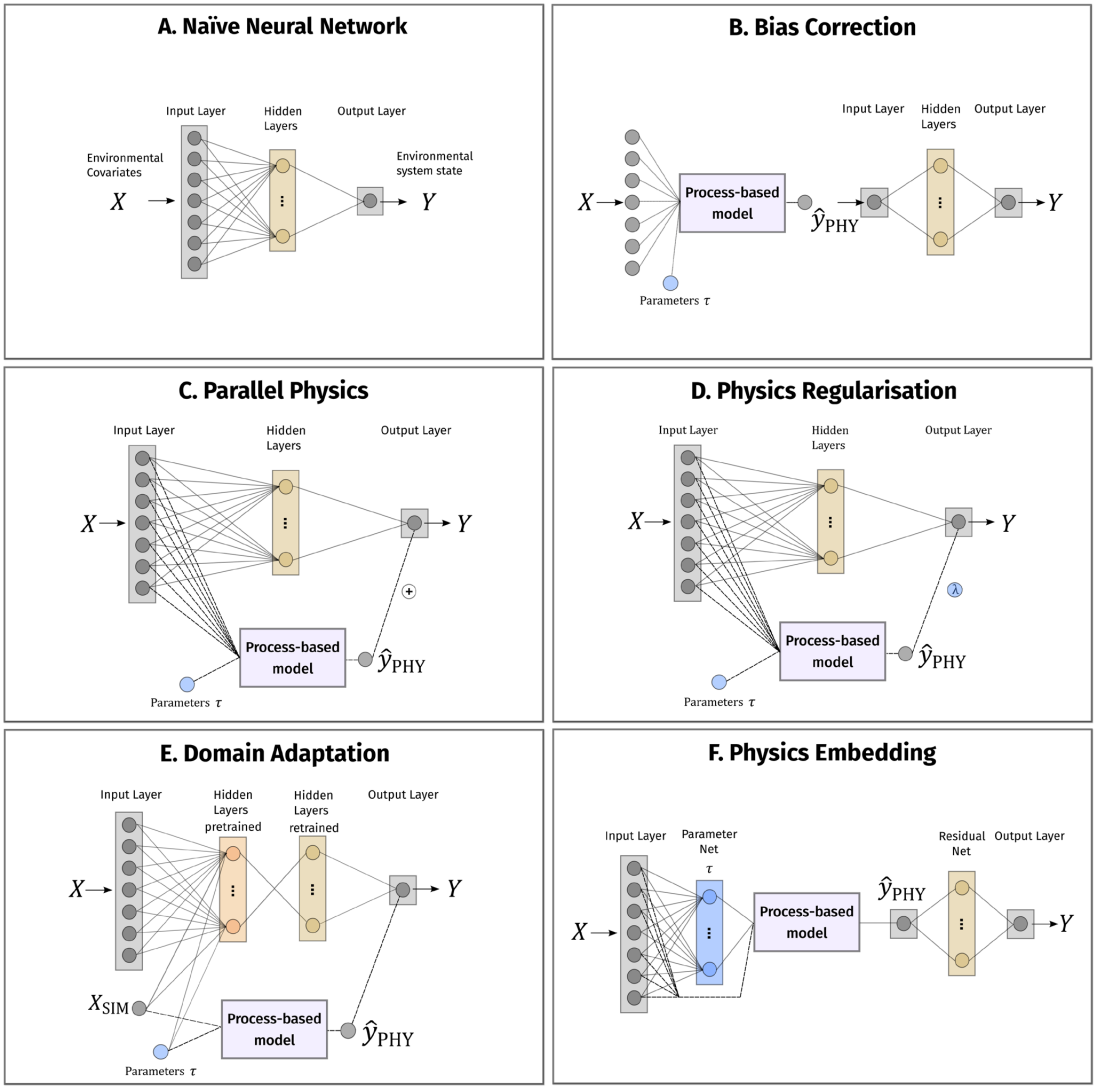
Schematic overview of the structure of five PINN approaches (B‐F) and a naïve neural network (A) for comparison. Blue elements mark model parameters of the process‐based model, grey mark data (environmental covariates X, model predictions Y and process predictions ${\hat{y}}_{\mathrm{PHY}}$). Note that the structure of physics regularisation is identical to that of the parallel physics, but the physics regularisation does not add process predictions to its own predictions. Instead, it evaluates both in a joint likelihood. Ellipses (…) indicate more layers and nodes, according to architecture and hyper‐parameter search. The physics embedding encodes the process‐based model as a forward pass in the neural network architecture and thus during training information of the gradients flows backwards through the process‐based model.

Physics regularisation is structurally similar to the parallel physics approach (Figure 2D) but cannot be rephrased as regression‐style problem. The NN maps environmental covariates X to the environmental state variable Y. PM predictions ${\hat{y}}_{\mathrm{PHY}}$ are used only in the loss function during training, such that the fitted NN minimises deviation from observation, while maintaining also minimal distance to PM predictions. Loss ℒ is calculated based on NN predictions ${\hat{y}}_{\mathrm{NN}}$, PM predictions ${\hat{y}}_{\mathrm{PHY}}$ and the observed y as

(3) $$ℒ={ℒ}_{\mathrm{MSE}}\left(y,{\hat{y}}_{\mathrm{NN}}\right)+\lambda \;{ℒ}_{\mathrm{MSE}}\left({\hat{y}}_{\mathrm{PHY}},{\hat{y}}_{\mathrm{NN}}\right),$$

with $\lambda \in \left(0,1\right]$ determining the strength of the regularisation and being defined in the hyper‐parameter search. The larger the λ, the stronger the predictions are tied to the PM.

### 2.5 Domain Adaptation

Domain adaptation differs structurally from the above approaches, as it consists of a specific training protocol instead of architectural modifications (Figure 2E): the NN is (i) pre‐trained on a rich and purely artificial dataset generated by the PM, yielding an emulator, followed by (ii) fine‐tuning the parameterised network by re‐training to the observations y (Gongora et al. 2019). The pre‐training dataset $X_{\mathrm{SIM}}$, $y_{\mathrm{SIM}}$ is generated by simulating $y_{\mathrm{SIM}}$ from the PM, based on an input similar to X, $X_{\mathrm{SIM}}$. Here, $X_{\mathrm{SIM}}$ is generated by generalised additive models describing X as a function of time. Parameters τ of the PM were sampled from their prior distributions in a Latin hypercube design. The number of inputs of the domain adaptation network is the same in pre‐ and re‐training. It takes environmental covariates as explicit inputs but ignores the parameter prior samples, as that information is represented in PM‐simulated $X_{\mathrm{SIM}}$ and $y_{\mathrm{SIM}}$ (for technical details, see Supporting Information).

### 2.6 Physics Embedding

Finally, the parameters of the PM can be turned from constants into functions of the input. This is what happens in ‘physics embedding’ (Figure 2F), where a parameter network represents the optimal parameters as a function of the model input, see also (Meng et al. 2021) for learning functional prior distributions with NN embedding instead of parameter point estimates. A bias correction re‐calibrates the PM output to fitted values. The key step of input‐driven, non‐constant PM parameters positions this approach closest to correlative modelling approaches: only the PM structure remains as a constraint but not its actual parameters.

The framework comprises three steps (Figure 2F). First, an multi‐layer perceptron (MLP)‐type ‘parameter network’ with multiple linear layers and ReLU activation maps environmental covariates X to the PM parameters τ. Second, the PM is run with the parameter estimates τ from this parameter network and the environmental covariates X. Third, the PM predictions ${\hat{y}}_{\mathrm{PHY}}$ are bias corrected with another MLP‐type network akin to the bias correction setup above, using all model outputs ${\hat{y}}_{\mathrm{PHY}}$ to estimate Y. In the loss function, the contribution of the PM to the overall loss is regularised (Equation 4) as

(4) $$ℒ={ℒ}_{\mathrm{MSE}}\left(y,{\hat{y}}_{\mathrm{NN}}\right)+\lambda \;{ℒ}_{\mathrm{MSE}}\left(y,{\hat{y}}_{\mathrm{PHY}}\right),$$

Parameter network and bias correction network are optimised simultaneously. Physics embedding dynamically estimate parameters for the PM and thus conceptually differs from the stand‐alone PM calibration. To correct for this additional flexibility towards the PM, we set the evaluation batch size to one, equivalently to the full time series. Parameter estimates are averaged over nodes and data points so that the PM receives only one value each, similar to the stand‐alone PM. Technically, the PM is defined as a forward pass connecting only the output layer of the parameter network and the input layer of the bias correction network. To guarantee gradient flow in the backpropagation through the PM, it needs to satisfy tensor compatibility in PyTorch.^1^ Thus, every elementary operation in the PM code was changed to its tensor equivalent.

BOX 1 **PINNs With Continuous Processes.**

We exemplify PINNs in a simulated scenario with an ODE PM. We use a Lotka–Volterra model with a sigmoidal feeding rate (type III functional response) to generate data and as PM a simplified version with a linear feeding rate (type I functional response). We test the predictive performance of PINNs under three conditions: (i) data sparsity, (ii) data sparsity + structural error in the PM and (iii) data sparsity + structural error in the PM + observation noise.

*Data‐generating model*. We consider the predator‐prey Lotka–Volterra system:

(5) $$\begin{array}{l} \frac{{\mathrm{dx}}_{t}}{\mathrm{dt}}={\mathrm{rx}}_{t}-b\frac{x_{t}^{2}y_{t}}{1+{\mathrm{sx}}_{t}^{2}},\\ \frac{{\mathrm{dy}}_{t}}{\mathrm{dt}}=b\frac{x_{t}^{2}y_{t}}{1+{\mathrm{sx}}_{t}^{2}}-{\mathrm{my}}_{t}, \end{array}$$

where r is the growth rate of prey, m is the mortality rate of predators and b is the feeding rate of predators described by a type III functional response with prey searching time s (Case 2000). We simulate the densities of predators and prey over 130 time units with initial densities $x_{0}=10,y_{0}=10$ and parameter values r=0.1,b=0.02,m=0.04,s=0.025.

*Observations*. We observe prey densities at nine random times. In (i)–(ii), the observed prey densities are used to calibrate the models. In (iii), we add Gaussian noise to the observed densities as $x_{t}+N\left(0,0.5\right)$.

*Process‐based model*. In (i), we assume that the PM exactly matches with the data‐generating model. In (ii, iii), we simplify the data‐generating model by changing the type III to the type I functional response. The PM is thus:

(6) $$\begin{array}{l} \frac{{\mathrm{dx}}_{t}}{\mathrm{dt}}={\mathrm{rx}}_{t}-{\mathrm{bx}}_{t}y_{t},\\ \frac{{\mathrm{dy}}_{t}}{\mathrm{dt}}={\mathrm{bx}}_{t}y_{t}-{\mathrm{my}}_{t}, \end{array}$$

assuming initial conditions $x_{0}=12,y_{0}=10$.

*PINNs*. We compare the performance of a parallel physics NN with the PM and an MLP. Both PINN and MLP condition $x_{t_{n}}$, the density of prey at observation n, on the previous observation 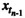 and times $t_{n-1},t_{n}$. For simplicity, both models use two hidden layers of size 50.

*Training and evaluation*. We train models in a leave‐one‐out cross‐validation with stochastic gradient descent (Kingma and Ba 2017) and mean squared error as loss function, using all data points in one batch. We train the PM for 60,000 epochs with learning rate $10^{-5}$, the MLP and the PINN for 200,000 epochs with learning rate $10^{-4}$. We evaluate each model on the full simulated time series (130 time units with time difference 0.5) and compute mean and standard error of predictions over the cross‐validation folds.

Results.

**FIGURE 3:**
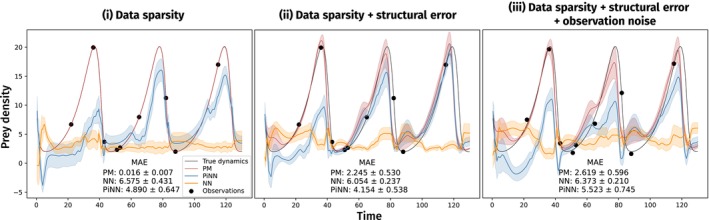
Comparison of evaluation predictions and mean absolute error (MAE) of the parallel physics (blue) with PM (red) and a deep NN (orange). Models were parameterised (i) under sparse observations, (ii) with additional structural error to the PM and (iii) with additional observation noise.

*Conclusion*. PINNs improve on the MLP under all conditions but remain inferior to the (approximately) true PM. Since in reality PMs may be structurally incorrect in multiple ways, the improvement relative to the MLP is the main advantage of PINNs (Figure 3).

## 3 Applying the Approaches

We tested the presented framework of physics‐informed neural networks (PINNs) in a case study focusing on three common ecological prediction problems using carbon and water fluxes from forest ecosystems measured using eddy covariance. The prediction problems were (i) temporal on‐site prediction, (ii) spatial multi‐site prediction and (iii) spatio‐temporal multi‐site prediction. For each problem, we considered two data availability scenarios: (A) rich data, using the pre‐processed daily time series, and (B) sparse data, where the time series from (A) was reduced to 1 day/week. For each combination of prediction problem and data scenario, we conducted separate parameter calibration, training, evaluation and post hoc variable importance analysis.

### 3.1 Naïve Neural Network

Neural networks (NN) do the mapping f:X↦Y through a hierarchically structured latent feature space, that is, hidden layers (Figure 2A). The latent space allows for the representation of hidden processes alongside the measured input X. Through layer‐specific transformations, that is, activation functions, the approximated function f can be highly nonlinear. The output of an NN with a single hidden layer is defined as

(7) $$y_{j}=\sigma \left(\left(\sum_{i}x_{i}\times W_{\mathrm{ji}}\right)+b_{j}\right),$$

where $y=\left\{y_{1},\ldots ,y_{j}\right\}\in Y$ is the j‐dimensional output vector and $x=\left\{x_{1},\ldots ,x_{i}\right\}\in X$ is the i‐dimensional input vector. The weight matrix $W_{\mathrm{ji}}$ and the bias vector $b_{j}$ are network parameters that are learned during the training procedure. The linear combination of inputs, weights and bias is transformed by the activation function σ, which is part of the hyper‐parameters of the network. Here, we used the most basic NN type, that is, a fully connected MLP. Through the choice of the linear activation function in the last layer and the mean squared error as the objective function, the MLP performs a regression task under the assumption of normality. Used without process guidance, this MLP is hereafter referred to as the naïve NN.

### 3.2 Process‐Based Model

The PM used to predict carbon and water fluxes in this case study is PRELES (PREdict Light‐use efficiency, Evapotranspiration and Soil water content) (Peltoniemi et al. 2015). PRELES is a semi‐empirical model that predicts gross primary productivity P, evapotranspiration E and soil water content θ at a daily time scale. More specifically, PRELES does the mapping f:X↦Y with $X=\left\{T,D,\phi ,R,f_{\text{aPPFD}},\left[{\mathrm{CO}}_{2}\right],d\right\}$ and $Y=\left\{P,E,\theta \right\}$, where T, D, ϕ, R, $f_{\text{aPPFD}}$, $\left[{\mathrm{CO}}_{2}\right]$ and d are air temperature (in Celsius), vapour pressure deficit (in kPa), photosynthetic active radiation (in mmol/m^2^), precipitation above the canopy (in mm), absorbed proportion of photosynthetic active radiation (−), (constant) atmospheric carbon dioxide concentration (in ppm) and day of the year, respectively. The mapping f consists of three coupled subsystems for P, E and θ. The calculation of θ is based on a three pool formulation, splitting θ into soil, surface and snow pools. The state of $\theta_{k}$ at day k depends on the state of the previous day, $\theta_{k-1}$. The subsystem for P‐prediction at a day k is based on a general model for light‐use efficiency (Mäkelä et al. 2008):

(8) $$P_{k}={\beta \phi }_{k}f_{\text{aPPFD}}\prod_{i}f_{i,k},$$

with β as the potential light‐use efficiency and $\prod_{i}f_{i,k}$ the product of exogenous‐driven modifiers for β. One of these modifiers, the soil water modifier $f_{W,P}$, links the subsystems of θ and P. In the third subsystem, E is calculated as directly depending on P. The subsystem is connected to θ by a modifier $f_{W,E}$. For more details on the PM structure, see (Peltoniemi et al. 2015).

### 3.3 Process‐Informed Neural Networks

In the section on methods overview, we proposed five different PINN‐like approaches that combine process knowledge with NNs (Figure 2). For our case studies, we constructed the PINNs such that they map environmental covariates, which the PM takes as input, $X=\left\{T,D,\phi ,R,f_{\text{aPPFD}},\left[{\mathrm{CO}}_{2}\right],d\right\}$, to the gross primary production P. Bias correction does not take these environmental covariates as input, but rather the PM predictions, in our case gross primary production, evapotranspiration and soil water content: ${\hat{y}}_{\mathrm{PHY}}=\left\{P,E,\theta \right\}$.

### 3.4 Study Sites and Data

We selected five forest sites from the PROFOUND database based on their data availability of environmental covariates and carbon turnover (Reyer et al. 2020). The forest sites cover deciduous, mixed and coniferous forest types with varying dominating species. The environmental covariates of the forest sites represent mediterranean, temperate and boreal climate. The PROFOUND database provides eddy‐covariance measurements from FLUXNET at a daily or half‐hourly resolution and satellite imagery data from MODIS at an 8‐day resolution. We chose variables and their resolution based on the PM requirements. Carbon turnover was measured as gross primary productivity GPP, P, that were taken from FLUXNET at daily resolution. The fraction of absorbed photosynthetic active radiation was taken from MODIS at 8‐day resolution and converted to daily resolution by assuming constant values within the 8‐day periods. The dataset was aligned in terms of resolution and units in a pre‐processing procedure (see Supporting Information), resulting in daily measurements of covariates and carbon turnover for five forest sites over four to 7 years. For the sparse dataset, we used only one data point per week, systematically selected from the full dataset for training.

### 3.5 Network Architecture Search

We selected NN architectures (number of hidden layers and nodes) and the parameters of the optimisation algorithm (learning rate and batch size) based on a combined random grid search. We first randomly drew architecture structures and parameter values and then tested their combination for each model. For physics regularisation, we additionally sampled the regularisation parameter λ. Only for the physics embedding network, the architecture was selected manually with λ for simplicity set to 1.

### 3.6 Training and Testing

#### 3.6.1 Neural Network

We split the data into training, validation and test set. We used training and validation data during the parametrisation procedure (training) and held out a test set of 1 year for evaluation. Training was done in a (temporally, spatially or spatio‐temporally, for case studies 1–3) blocked cross‐validation (Roberts et al. 2017) such that the models could be validated for a full year in each fold. The test year 2008 was always held out from the cross‐validation and only used to evaluate the models for their predictive error and uncertainty (Bates, Hastie, and Tibshirani 2024). Models are trained for 5000 epochs with the Adam optimisation algorithm (Kingma and Ba 2017).

#### 3.6.2 Process‐Based Model

We calibrated 13 model parameters in a block‐cross‐validation using an MCMC algorithm with a DREAMZ sampler (Vrugt et al. 2009) over 50,000 iterations and three chains, using the R‐package BayesianTools (Hartig, Minunno, and Paul 2019). Following previous works that conducted model calibrations of forest productivity (Rödig et al. 2017; Minunno et al. 2019; Stocker et al. 2020), and based on the assumption of normality in the machine learning models (Liu et al. 2016; Montero et al. 2024), we used a Gaussian likelihood function with model predictions as mean and a standard deviation of one (ter Braak and Vrugt 2008), even though being aware of the strength and limitations of this assumption.

### 3.7 Inference

We conducted a first step into inference by evaluating trained models in a post hoc variable importance analysis (Ryo et al. 2021; Molnar 2022). We computed individual conditional expectations by varying explanatory variables separately over a predefined range. For each model and each varying explanatory variable, we predicted daily GPP values for four 2‐week periods that include the following days in March (representing spring), June (representing summer), September (representing autumn) and December (representing winter). The average over this 2‐week period displays the partial dependence of the model predictions on each climatic variable.

## 4 Prediction Problem 1

### 4.1 Prediction Problem 1: Same Site, Other Year

**FIGURE 4:**
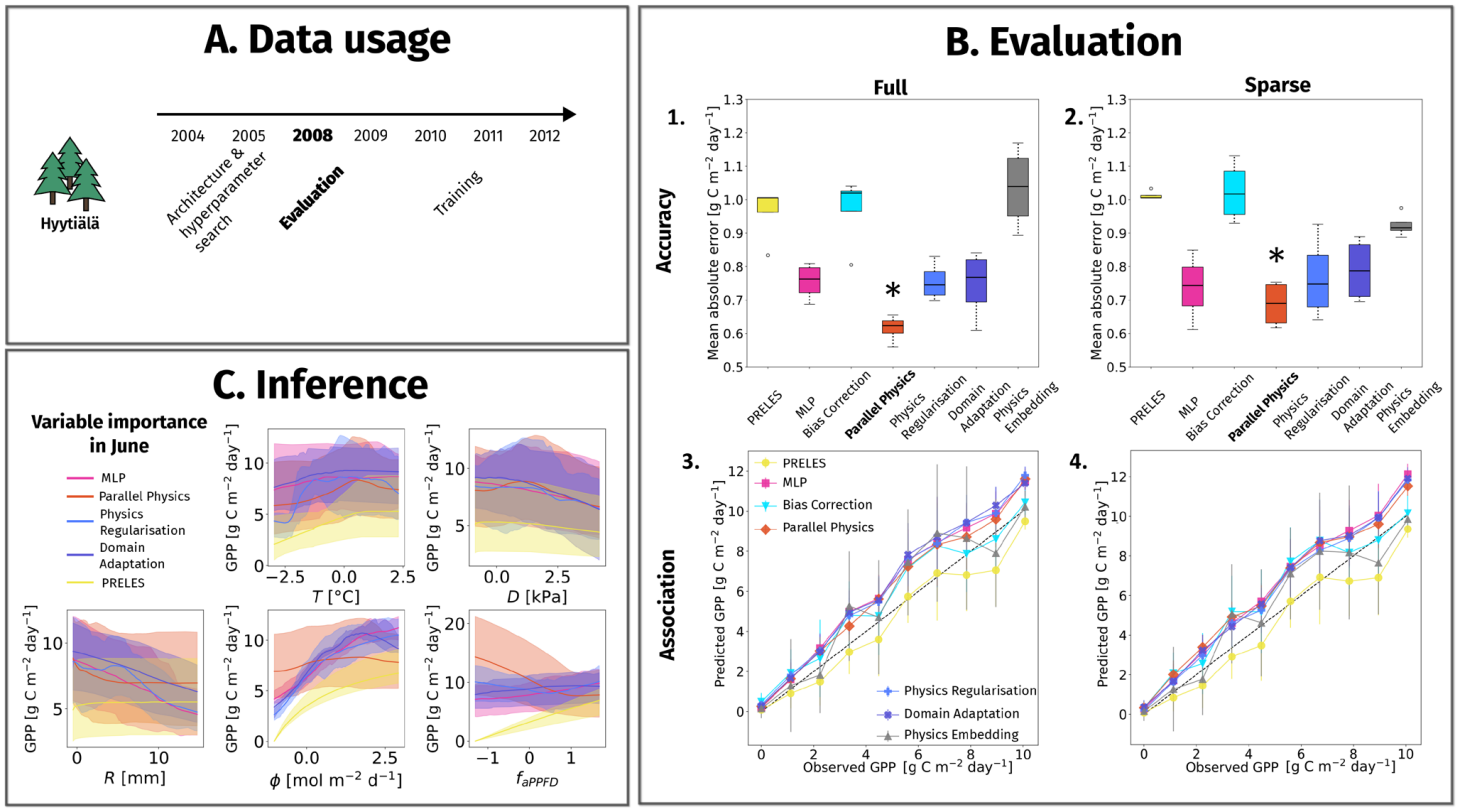
Overview of (A) the data usage in the temporal analysis, (B) predictive performance for gross primary productivity of PINNs compared to a PM and an MLP in accuracy (mean absolute error) (1 and 2) and in quantile association (3 and 4), and (C) the post hoc variable importance analysis in during growing season in a 2 week period in June for temperature (T), vapour pressure deficit (D), precipitation (R), photosynthetic active radiation (ϕ) and the fraction of absorbed photosynthetic active radiation ($f_{\text{aPPFD}}$). The models with best performance in accuracy are marked in bold and with an asterisk in (B).

We evaluated the predictive performance of PINNs in temporal extrapolation, relative to the PM and MLP. We used time series data from the forest stand Hyytiälä, at which PRELES was developed. We used the years 2004, 2005 for the network architecture search and the years 2009–2012 for training and PM model calibration. We evaluated the performance of all models in the year 2008 (Figure 4A).

All models predicted GPP as accurately as or better than PRELES (Figure 4B1,2), except the bias correction network, and the physics embedding network in full data scenario. The naïve network was outperformed only by the parallel physics network. Relative performances comparing sparse and full data scenario were consistent. Errors increased with magnitude for almost all models, and the GPP was slightly overestimated (Figure 4B3,4). Inference using conditional variable importance analysis allowed us to compare variable effect shapes and sizes between models (Figure 4C). The visual analysis implied that photosynthetically active radiation ϕ was the most important variable: the effect was small in PRELES but well developed in all PINNs. For precipitation R, all PINNs showed stronger effect sizes than PRELES. The regularised PINN was sensitive to low temperatures.

### 4.2 Prediction Problem 2: Same Year, Other Site

**FIGURE 5:**
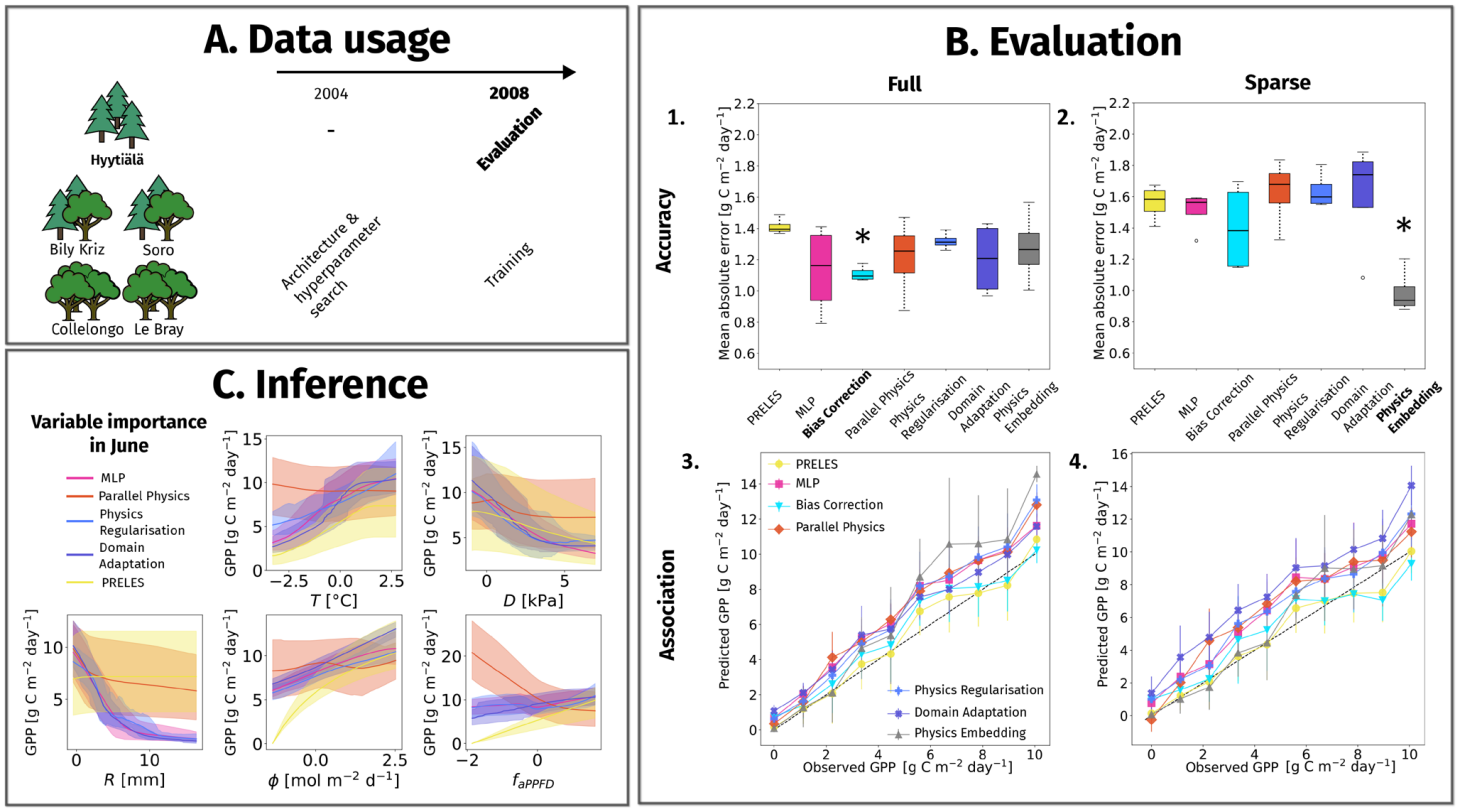
Overview of (A) the data usage in the spatial analysis, (B) predictive performance for gross primary productivity of PINNs compared to a PM and an MLP in accuracy (mean absolute error) (1 and 2) and in quantile association (3 and 4), and (C) the post hoc variable importance analysis in during growing season in a 2‐week period in June for temperature (T), vapour pressure deficit (D), precipitation (R), photosynthetic active radiation (ϕ) and the fraction of absorbed photosynthetic active radiation ($f_{\text{aPPFD}}$). The models with best performance in accuracy are marked in bold and with an asterisk in (B).

We evaluated the predictive performance of PINNs in spatial extrapolation, relative to the PM and MLP. We used data from the forest stands Hyytiälä, Bily Kriz, Collelongo, Solling and Le Bray, which differ substantially in climate and forest composition. We used time series data of the year 2004 for all forest sites for the network architecture search. Models were trained on the years 2005 and 2008 of all sites but Hyytiälä and evaluated on data of 2008 for Hyytiälä (Figure 5A).

Accuracy decreased generally compared to prediction problem 1, except for the physics embedding network that performed similarly in spatial and temporal predictions (compare Figures 4B1,2 and 5B1,2). The bias correction outperformed the MLP under full and the physics embedding network under sparse data setting. Most models overpredicted GPP, aside from bias correction and physics embedding (Figure 5B3,4) which were both well correlated with PRELES predictions in lower and higher ranges. The results of best accuracy and association scores aligned: bias correction and physics embedding had lowest errors in both evaluation approaches.

For prediction problem 2, the visual variable importance analysis implies higher sensitivity of all NNs to climatic predictors (Figure 5C). While their trends remained broadly the same, the PINNs differed stronger from PRELES in effect shape and size. Overall, the PINNs showed higher sensitivity for R and less for ϕ, compared to PRELES. Furthermore, for ϕ, effect curves for the PINNs remained clear but became linear, while their non‐linearity in R increased relative to the temporal prediction.

### 4.3 Prediction Problem 3: Other Site, Other Year

**FIGURE 6:**
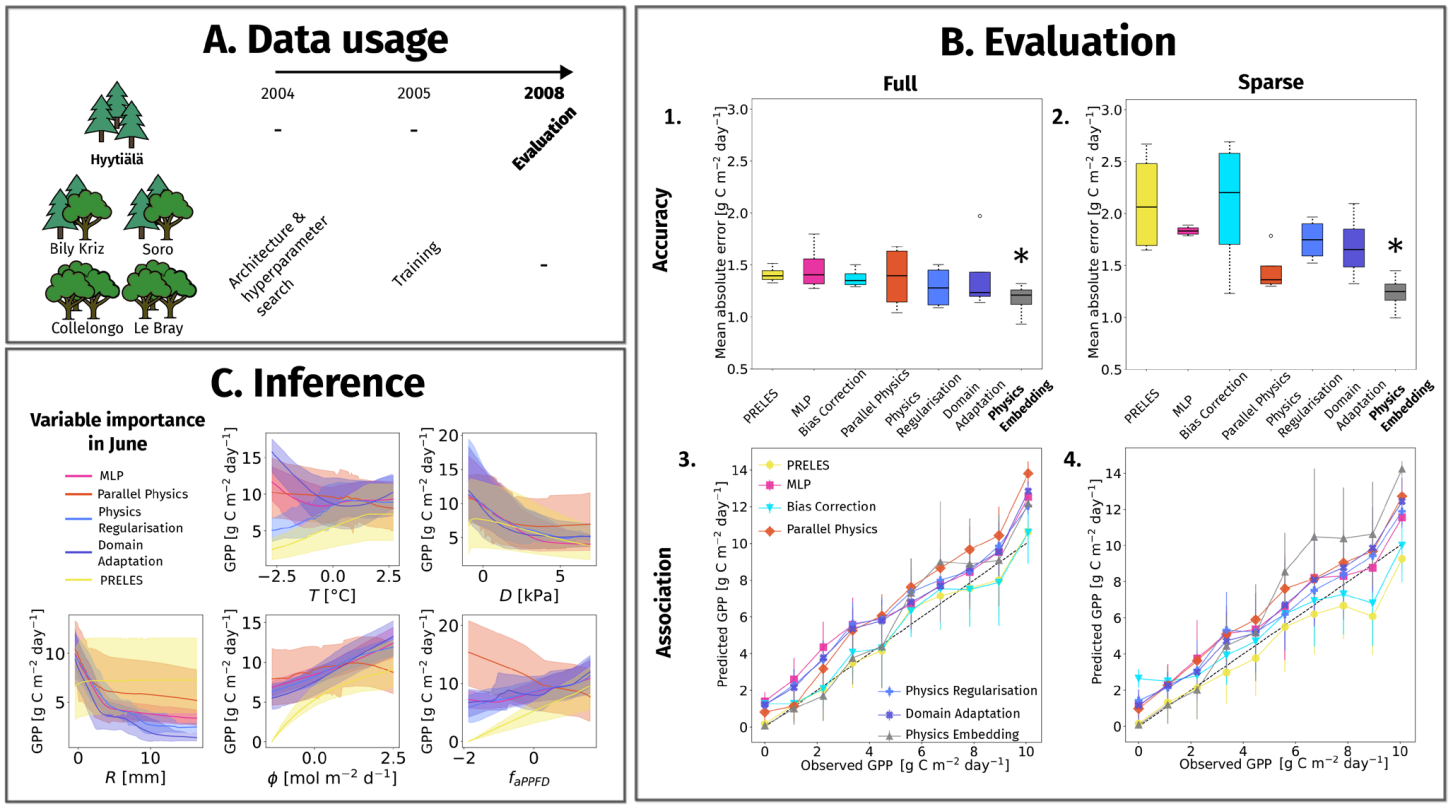
Overview of (A) the data usage in the spatio‐temporal analysis, (B) predictive performance for gross primary productivity of PINNs compared to a PM and an MLP in accuracy (mean absolute error) (1 and 2) and in quantile association (3 and 4), and (C) the post hoc variable importance analysis in during growing season in a 2‐week period in June for temperature (T), vapour pressure deficit (D), precipitation (R), photosynthetic active radiation (ϕ) and the fraction of absorbed photosynthetic active radiation ($f_{\text{aPPFD}}$). The models with best performance in accuracy are marked in bold and with an asterisk in (B).

We evaluated the predictive performance of PINNs in spatio‐temporal extrapolation, relative to the PM and MLP. We used data from the forest stands Hyytiälä, Bily Kriz, Collelongo, Solling and Le Bray and employed the same network architectures and parameters as in prediction problem 2. We trained the models on time series data of the year 2005 for all forest stands but Hyytiälä and evaluated the models on data of the year 2008 and site Hyytiälä (Figure 6A).

PRELES, the naïve and the bias correction network performed similarly accurate (Figure 6B1,2). All three showed a clear deterioration from the full to the sparse data setting. The other PINNs improved slightly on the naïve network and PRELES, while predictive error bars overlap. An improved performance of all PINNs but the bias correction could be detected in the sparse data setting. In both data availability scenarios, the physics embedded network performed best, showing a similar magnitude of accuracy for full and sparse data availability. Bias correction and physics embedding network again correlated best with PRELES in the full and sparse data setting. In all three case studies, some cross‐validation folds caused high variance in accuracy. These uncertainties increased from the full to the sparse data setting. We further note a stronger overestimation of GPP in the winter season for almost all PINNs (Figure 6B3,4). PRELES generally underestimated GPP for high production values, especially under sparse data availability.

In the variable importance analysis, we found trends and effect sizes very similar to those of prediction problem 2, except for temperature, where PINN effect curves displayed stronger non‐linearity (Figure 6C). In the case of ϕ, the parallel physics network corrected PRELES predictions downwards in high ranges, implicitly indicated by the negative slope. This was similar to prediction problem 1, while a slightly positive correction was visible in prediction problem 2. Looking at the full prediction over 2008 (Figure 6C), the MLP and the PINN (Figure 6C parallel physics network) showed a slightly positive bias outside the growth season.

## 5 Discussion

We developed and compared five process‐informed neural networks (PINNs) that integrate data‐driven learning with process theory. For the example of carbon and water fluxes in forest ecosystems, the predictive performance of the PINNs was evaluated along a gradient of extrapolation and data availability. The relevance of PINNs is founded in three classical problems in process‐oriented environmental and ecological modelling: (i) low data availability, which limits options for process‐based model (PM) calibration and the use of deep neural networks (NNs) (Reichstein et al. 2019); (ii) limited process understanding, which manifests itself as structural mis‐specification in PMs (Wood 2001); and (iii) complex ecosystems, limiting the transferability of PMs (Yates et al. 2018). The approaches we presented complement a long‐existing and large body of works that integrate correlative and PMs in a Bayesian hierarchical framework (Wikle and Hooten 2010; Hefley, Broms et al. 2017; Hefley, Hooten et al. 2017) by a deep‐learning perspective.

In each of our three prediction problems, at least one of the five PINN approaches showed the potential to outperform both the PM and the naïve NN in predictive accuracy. Our systematic evaluation of PINNs suggests that they may be preferable to a stand‐alone PM or NN with increasing complexity of the modelling task, in our case, especially in a sparse multi‐site spatio‐temporal transfer task (Figure 6). The PINNs' power is most notable when these approaches are combined, and the PM is fully integrating into the NNs' architecture, as in ‘physics embedding’. The post hoc variable importance analysis used to interpret the PINNs process representation (Molnar 2022) suggests their similar sensitivities to climatic predictors.

### 5.1 Process Information for Better Predictions

Each prediction problem indicated that PINNs can improve predictive performance towards a stand‐alone naïve NN and a PM (Figures 4, 5, 6). PINN performs best varied with the complexity of the problem and data availability (Figure 1). Contrary to the results of the spatial and spatio‐temporal experiment, in the temporal experiment the more flexible models outperformed the more constrained models (Figure 4B). Especially the high performance of ‘parallel physics’ indicates the potential of explaining PM residuals by environmental covariates. In the spatial experiment, where PM predictions show a systematic bias, the simple ‘bias correction’ outperformed the other models. Such ability of NNs in post‐processing of systematic errors has been shown in ensemble weather forecasting (Rasp and Lerch 2018). Physics embedding combines dynamic PM parametrisation with different PINN approaches (regularisation and bias correction). It performed best in the most complex tested case with spatio‐temporal prediction under low data availability. Related works have shown the potential of this approach, for example, when parametrising ODEs from highly structured data (Köber et al. 2022) or parametrising light‐use efficiency models from different forest stands (Bao et al. 2023), and our results suggest they should be investigated more widely, despite their high computational costs. While the required tensor compatibility in PyTorch may limit full integration of large numerical models with NNs, JAX as an alternative framework combines numpy with automatic differentiation (Frostig, Johnson, and Leary 2018), the usage of which we demonstrate alongside our showcase in Box 1 (see Methods section).

### 5.2 Process Information for Better Transferability

A prominent motivation for the integration of PMs and data‐driven approaches is the latter's expected low transferability under new conditions (Pichler and Hartig 2023a; Karlbauer et al. 2022). In our most difficult transfer experiments, the spatial and spatio‐temporal prediction, naïve NNs exhibited a systematic bias of under‐prediction of primary production in the non‐growing season (see Supporting Information). We interpret this as an inability of PRELES to represent both deciduous tree‐dominated summer GPP and coniferous tree‐dominated winter GPP. Compared to the NN, the PM captures shared across‐site processes only in the model parameters, whereas physical relationships between parameters, climate data and environmental state variables remain the same.

In our spatial and spatio‐temporal experiments, the PM parameters showed higher variances compared to the on‐site prediction experiment (see Supporting Information), which could mean that shared across‐site processes remain misrepresented, undetected and unlearned from the observational data in the PM calibration. This is not unexpected when adapting a model to data and also not a critical problem when the goal is prediction (Hefley, Broms et al. 2017); however, for more general application, such process ideally would form part of the PM. PINNs are more flexible than the PM to learn and represent shared across‐site processes from the observational data while additionally being constrained with site‐specific process knowledge.

In the more flexible and less constrained PINNs, we see biased predictions in the high‐transfer experiments (prediction problems 2 and 3). The more constrained ‘parallel physics’ and especially the most constrained ‘physics embedding’ correct for this bias. Thus, we expect that with high‐transfer tasks, where training and evaluation data do not share all physical relationships, constraining an NN with physiological knowledge is especially powerful.

### 5.3 Process Information for the Inference of Undetected Processes

One drawback in the application of deep NNs and also of integrated approaches is the poor interpretability of the fitted model parameters and the causes of specific predictions. The field of explainable machine learning is just emerging (Pichler and Hartig 2023b), and model‐agnostic tools applicable for regression models can also be applied to NNs and PINNs (Molnar 2022; Ras et al. 2022). The visual conditional variable importance analysis allows us to compare size, trend and shape of sensitivities between models. In our analysis, photosynthetic active radiation ϕ and its absorbed fraction $f_{\text{aPPFD}}$ showed highest sensitivities for predicted gross primary production across all models, reflecting the PM's foundation in a light‐use efficiency model (Peltoniemi et al. 2015).

The PINNs' sensitivity to precipitation increases over the complexity of the transfer task from temporal to spatio‐temporal extrapolation, a trend we interpret as more and more correction of the simplistic light‐use efficiency model when data allow the NN to do so. Especially the parallel physics network, which learns the PM error as function of PM inputs, reduces the PM's sensitivity to precipitation. Thus, we conclude that for general use in different forest ecosystems PRELES might be too sensitive to precipitation. However, we cannot causally draw the conclusion that the PM is mis‐specified, for which further tests are needed.

Nevertheless, our variable importance analysis indicates trends of corrections and new sensitivities compared to the PM, which highlights the potential of PINNs for gaining process understanding. This global and exploratory inference approach could well be extended by local interpretable model‐agnostic explanations or Shapley values (Ryo et al. 2021).

### 5.4 Conclusion

We systematically developed and compared five process‐informed neural networks to investigate their suitability for environmental and ecological modelling tasks and beyond. Individual PINNs can improve results in ecological and environmental prediction experiments along the data availability and transfer task spectrum. This line of research is developing fast in physical applications and for differential equations (Karlbauer et al. 2022; Raissi, Perdikaris, and Karniadakis 2019; Karniadakis et al. 2021). We have presented here the potential applicability of PINNs for ecosystem flux prediction as an example but see their potential for all ecological research where mechanistic understanding is available, yet structurally incomplete. Combining PMs with neural networks may allow for better interpretability of the neural network component by addressing mis‐specification in the PM.

While we assessed the predictive capabilities of PINNs, their suitability and optimal choice depend on the specific problem. A dynamic physics‐informed neural network (PINN) calibration (Meng et al. 2021), that is, physics embedding, might be valuable when high computational resources are available and accuracy is needed. When both process‐model simulations and observations are available, the cost‐effectiveness of parallel and regularised physics makes them a good benchmark. Similarly, a bias correction method is useful for correcting errors in model predictions. Pre‐training a neural network on a large but not identical dataset, that is, domain adapting, can improve accuracy with sparse data and reduce computational costs (Erhan et al. 2010). Overall, the PINN approaches offer a complementary tool to stand‐alone mechanistic or correlative models, suitable for various stages of model development.

## Author Contributions

M.W., N.M. and C.F.D. conceived the study. M.W. and N.M. coded the neural network‐process model combinations and ran the fitting and prediction. M.K. and J.B. provided valuable comments. M.W., N.M. and C.F.D. analysed the results and wrote the manuscript.

### Peer Review

The peer review history for this article is available at https://www.webofscience.com/api/gateway/wos/peer‐review/10.1111/ele.70012.

## Supporting information


Data S1.

## Data Availability

The code for the analyses can be found here: https://github.com/biometry/PiNNs. Data, results and models are available here: https://osf.io/7gzbn/?view_only=99712c8233b6429d881556ec4cecc37c.

## Endnotes

^1^

https://pytorch.org/docs/stable/index.html.
