# Supplementary material for: Process‐Informed Neural Networks: A Hybrid Modelling Approach to Improve Predictive Performance and Inference of Neural Networks in Ecology and Beyond
Source: Ecol Lett. 2024 Dec 3;27(11):e70012. doi: 10.1111/ele.70012 (PMC11613309; doi:10.1111/ele.70012)
Supplement: Supplementary file 1 — Data S1. [file ELE-27-0-s001.zip › CaseStudy1.pdf]

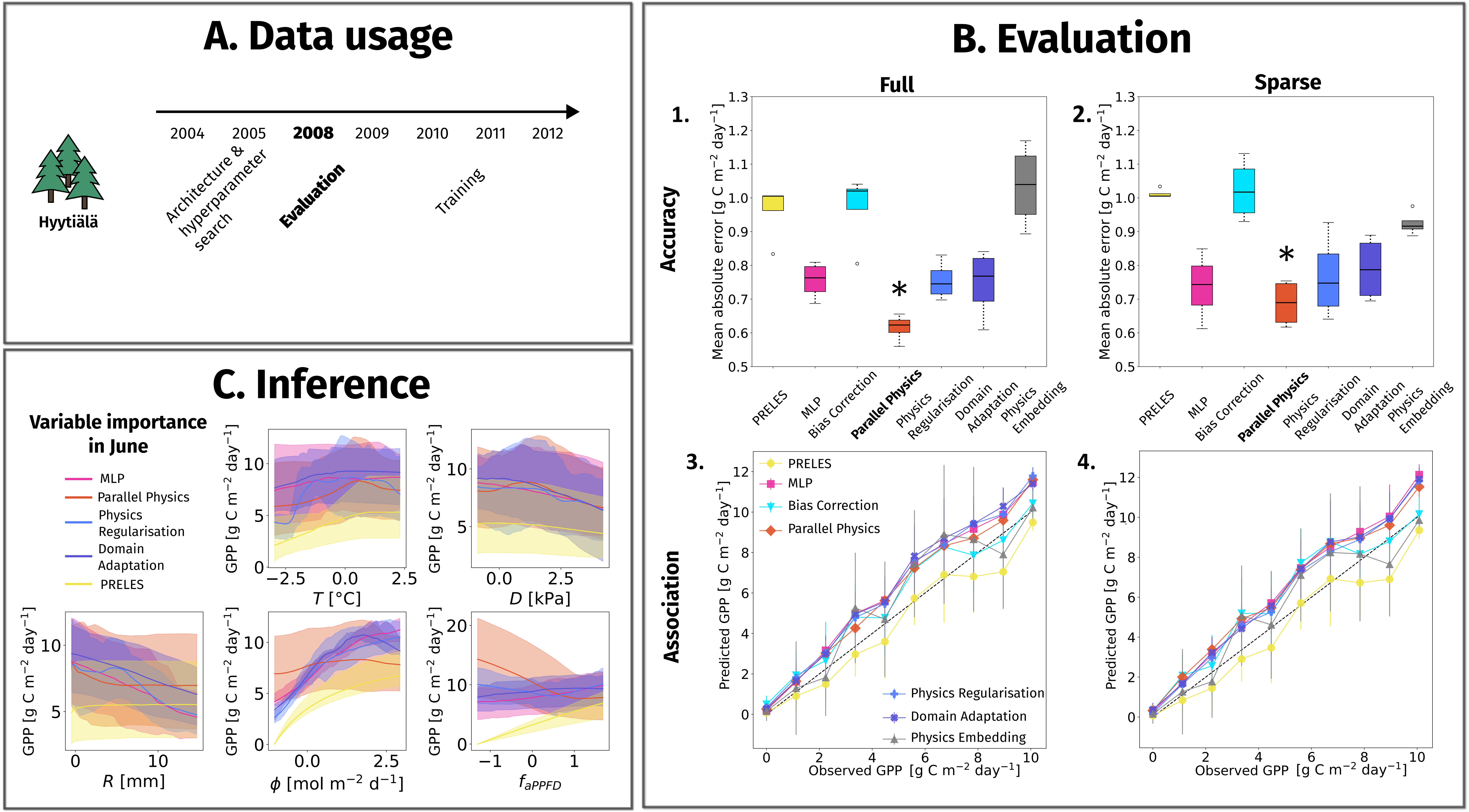
A. Data usage
2004
2005
2008
2009
2010
2011
2012
Hyytiälä
Architecture & hyperparameter search
Evaluation
Training
B. Evaluation
Full
Sparse
1.
2.
Accuracy
Mean absolute error [g C m^{-2} day^{-1}]
PRELES
MLP
Bias Correction
Parallel Physics
Physics Regularisation
Domain Adaptation
Physics Embedding
3.
4.
Association
Predicted GPP [g C m^{-2} day^{-1}]
Observed GPP [g C m^{-2} day^{-1}]
C. Inference
Variable importance in June
GPP [g C m^{-2} day^{-1}]
T [°C]
D [kPa]
R [mm]
ϕ [mol m^{-2} d^{-1}]
f_{aPPFD}
