# Supplementary material for: Process‐Informed Neural Networks: A Hybrid Modelling Approach to Improve Predictive Performance and Inference of Neural Networks in Ecology and Beyond
Source: Ecol Lett. 2024 Dec 3;27(11):e70012. doi: 10.1111/ele.70012 (PMC11613309; doi:10.1111/ele.70012)
Supplement: Supplementary file 1 — Data S1. [file ELE-27-0-s001.zip › SI.pdf]

Supplementary material

Process-informed neural networks: a hybrid modelling approach to improve predictive performance and inference of neural networks in ecology and beyond.

1 Pipeline **3**

1.1 Structure 3

1.2 Download code 3

1.3 Reproducibility 5

2 Data **5**

2.1 Source 5

2.2 Preprocessing 5

2.3 Normalisation 7

3 Technical details **8**

3.1 Domain adaptation 8

3.2 Physics embedding 9

4 Case studies **10**

4.1 Case study 1: temporal on-site prediction 10

4.1.1 Network architecture and hyperparameter search 10

4.1.2 Bayesian Calibration of the process model 10

4.1.3 Evaluation 12

4.1.4 Inference 12

4.2 Case study 2: spatial multi-site prediction 17

4.2.1 Network architecture and hyperparameter search 17

4.2.2 Bayesian Calibration of the process model 17

4.2.3 Evaluation 17

4.2.4 Inference 20

4.3 Case study 3: spatio-temporal prediction 20

4.3.1 Network architecture and hyperparameter search 20

4.3.2 Bayesian Calibration of the process model 20

4.3.3 Evaluation 22

4.3.4 Inference 22

1 Pipeline

1.1 Structure

The pipeline structure is sketched in Fig. 1.

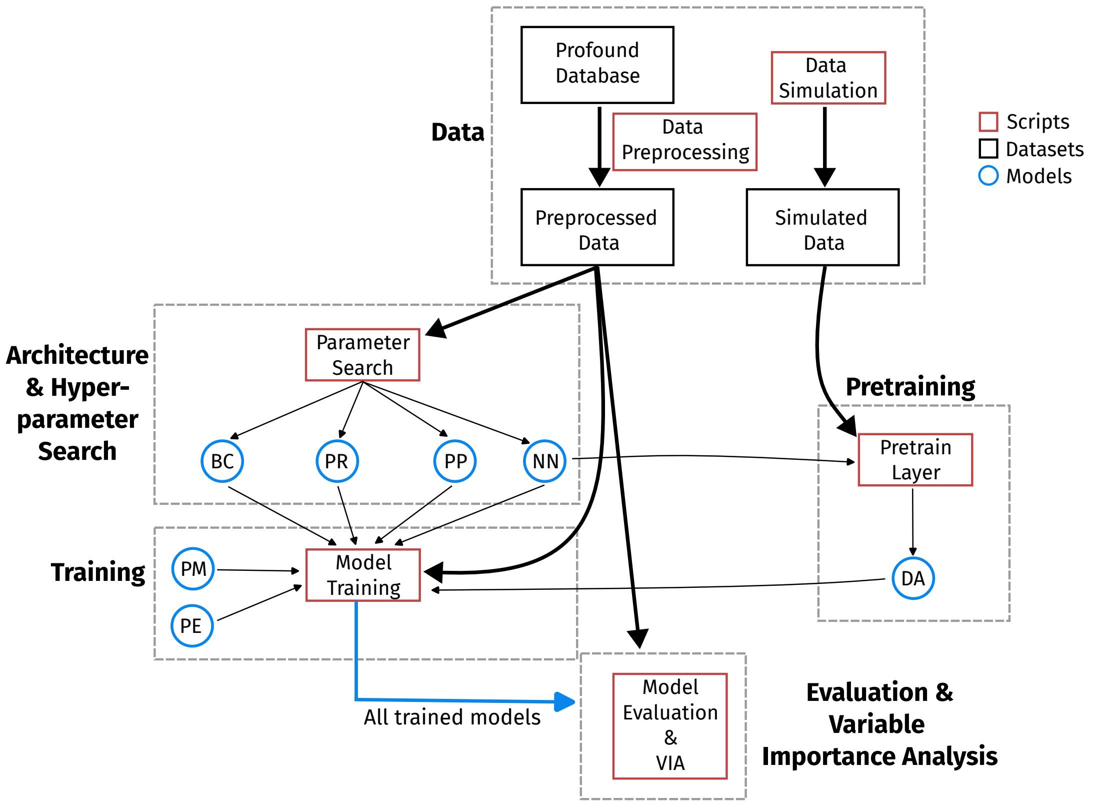

Figure 1: Sketch of the pipeline structure. The sequential order of the parts is from top to bottom. The structure is similar for the temporal, spatial and spatio-temporal experiment. PM = Process-based model, BC = Bias correction, PR = Physics Regularisation, PP = Parallel physics, NN = Naive neural network, DA = Domain adaptation, PE = Physics Embedding

1.2 Download code

The code pipeline can be downloaded from GitHub `https://github.com/biometry/PiNNs`.

The pipeline consists of the folders src, data, code and r. The modified source code of PRELES is stored in the src folder. To compile the modified PRELES code to a PyTorch cpp-extension, that is callable from Python the source code needs to be compiled. We use the compiler version that is shown below.

```
niklas@hpc: $ gcc --version
gcc (GCC) 9.1.0
Copyright (C) 2019 Free Software Foundation, Inc.
This is free software; see the source for copying conditions.  There is NO
warranty; not even for MERCHANTABILITY or FITNESS FOR A PARTICULAR PURPOSE.
```

To convert the modified PRELES c-code to a proper PyTorch cpp-extension run the setup.py script in the src folder using the command:

```
niklas@hpc: $ python setup.py install
running install
running bdistegg
running egginfo
writing preles.egg-info/PKG-INFO
writing dependencylinks to preles.egg-info/dependencylinks.txt
writing top-level names to preles.egg-info/toplevel.txt
reading manifest file 'preles.egg-info/SOURCES.txt'
writing manifest file 'preles.egg-info/SOURCES.txt'
```

```
installing library code to build/bdist.linux-x8664/egg

running installlib

running buildext

building ’preles’ extension

...

Processing dependencies for preles==0.0.0

Finished processing dependencies for preles==0.0.0
```

After running the setup script one can test if PRELES is callable in Python by the commands below. To call PRELES it is necessary to import torch first.

```
niklas@hpc : $ python

Python 3.7.10 — packaged by conda-forge — (default, Feb 19 2021, 16:07:37)

[GCC 9.3.0] on linux

Type ”help”, ”copyright”, ”credits” or ”license” for more information.


import torch

import preles
```

1.3 Reproducibility

2 Data

2.1 Source

We use the PROFOUND database. It provides information about the vegetation and climate at the forest stand scale in time series consisting of data of several years for forest sites in Europe?. The PROFOUND database combines empirical data about forest stand structure, species composition, climatic conditions, energy and mineral fluxes. The data can be downloaded from the GitHub `https://github.com/COST-FP1304-PROFOUND/ProfoundData`.

2.2 Preprocessing

For the analysis, the same input variables are used in the same units as in PRELES. The variables needed are namely the daily sums of photosynthetic active radiation (PAR), the mean air temperature (TAir), the vapour pressure deficit (VPD), the precipitation above canopy (Precip), the carbon dioxide concentration of air (CO_2), the fraction of photosynthetic active radiation (fAPAR) and the day of year (DOY), as depicted in Table 5. Additionally, information on the gross primary production (GPP) is required. It is used as the output variable in the models. The data required is in a daily resolution.

The PROFOUND database information on the irradiance, the global radiation ϕ_e (in $\mathrm{J\,cm^{-2}\,d^{-1}}$) is converted into the quantum units describing the photon irradiance ϕ_p (in $\mathrm{mol\,m^{-2}\,d^{-1}}$). Therefore, the relation of the wavelength and the energy of a photon is used

Table 1: Data used from the PROFOUND database.

Abbreviation	Description	Unit
PAR	sum of photosynthetic active radiation	$\mathrm{mol\,m^{-2}\,d^{-1}}$
TAir	mean air temperature	°C
VPD	mean vapour pressure deficit	kPa
Precip	precipitation above canopy	mm
CO_2	carbon dioxide concentration	ppm
fAPAR	fraction of photosynthetic active radiation	-
GPP	gross primary production	$\mathrm{g\,C\,m^{-2}\,d^{-1}}$

as described in (**?**) with

$$\phi_{\mathrm{p}} = \frac{\phi_{\mathrm{e}}}{E N_{\mathrm{A}}} \quad , \tag{1}$$

and

$$E = \frac{hc}{\lambda} \quad , \tag{2}$$

where h is Planck's constant ($6.63 \cdot 10^{-34}\mathrm{J\,s}$), c is the speed of light ($2.99792458 \cdot 10^{8}\mathrm{m\,s^{-1}}$), λ is the wavelength ($\approx 2.2{\cdot}10^{-7}\mathrm{m}$), N_{A} is Avogadro's constant ($6.602{\cdot}10^{23}\mathrm{mol^{-1}}$) and ϕ_{e} is the global radiation (in $\mathrm{J\,s^{-1}\,m^{-2}}$).

fAPAR is derived from MODIS satellite data and has a resolution of eight days. The data gaps of the eight day resolution data are filled by averaging information of the data point before and after the gap. Second, to get the daily resolution, it is assumed that the eight day

value is representative for all days within this period. Therefore, fAPAR is set constant over the eight days.

GPP is converted from μmol CO_2 m^{-2} s^{-1} to g C m^{-2} d^{-1} using the molar mass of carbon ($\approx 12.011 g\,mol^{-1}$).

2.3 Normalisation

Each variable is normalised around its mean σ and its standard deviation σ before using it as an input in the neural networks. Therefore, a z-score is calculated for each variable as

$$z = \frac{x - \mu}{\sigma} \quad . \tag{3}$$

Because of its cyclic character a sine and cosine function is used to normalise DOY (d). This ensures that the information of the last day of year n and the first day of year $n + 1$ are closer together (compared to 365 and 1). DOY is transformed as

$$d_{\text{sin}} = \sin\left(d\frac{2\pi}{365}\right) \quad , \tag{4}$$

and

$$d_{\text{cos}} = \cos\left(d\frac{2\pi}{365}\right) \quad . \tag{5}$$

3 Technical details

3.1 Domain adaptation

The yearly equivalents of the neural network training data were the basis for the climate simulations. Except for CO_2, which was fixed at a value of 380 ppm, the five climatic vari-

ables $y = \{\text{T}, \phi, \text{D}, \text{P}, f_{\text{aPPFD}}\}$ were separately modeled with a generalized additive model (GAM). For the on-site simulations, each $y_k \in y$ was described as a function of the day of the year (DOY) in interaction with the year R. The GAM was fitted with a cyclic cubic smooth function f imposed onto DOY. Allowing for the interaction with R, the smooth varies with the year.

$$y_k = \beta_0 + \sum_{i=1}^{4} f_i(\text{DOY}) \cdot R_i + \epsilon \tag{6}$$

For the multi-site simulations, each y_k was described as a function of the day of the year (DOY) in interaction with the site S.

$$y_k = \beta_0 + \sum_{i=1}^{5} f_i(\text{DOY}) \cdot S_i + \epsilon \tag{7}$$

The resulting models could be used to simulate estimated time series of the variables of any length and for any time point of years and sites under consideration. To introduce variation to the simulation, noise ν was added to each $\hat{y}_k$, sampled from a multivariate normal distribution $\nu \sim \mathcal{N}_{k=5}(\mu, \Sigma)$. Here, μ denotes the k-dimensional mean vector and Σ is the $k \times k$-dimensional covariance matrix. Means μ were set to zeros while the covariance matrix Σ was specified explicitly as the covariance matrix of the k-dimensional residual matrix.

With both, a global and local sensitivity analyses, the five most sensititive stand specific PRELES parameters were considered. These are the potential light use efficiency (β), the threshold for the state of acclimation ($X0$), the light modifier parameter for saturation with irradiance (γ), and the transpiration and evaporation parameters (α and χ). Prior knowledge about their marginal distributions is available as uniform prior distribution parameters, used

for the Bayesian calibration of PRELES (?). In order to provide the neural network with the same information as PRELES, the prior distributions were strongly narrowed down, ressembling the default calibration. In a Latin hypercube design the five parameters were sampled from their neat uniform joint probability distribution. The other parameters remained fix at their default values.

3.2 Physics embedding

We convert the PRELES c-code into a callable Python library. Therefore, we change the elementary operations in the c-code to their PyTorch equivalent, e.g. `sin(x)`→`torch.sin(x)`. This is a necessary condition to use PRELES as a forward pass of the neural network. In the PyTorch framework all calculations on the input tensor are stored in a computational graph during forward propagation. During backpropagation the computational graph of the tensor is used to numerically calculate intermediate gradients for all elementary operations on the tensor.

4 Case studies

4.1 Case study 1: temporal on-site prediction

4.1.1 Network architecture and hyperparameter search

We implemented a combined architecture and hyper-parameter search. The architecture search space consists of 300 randomly sampled layersizes with a maximum depth of 4 hid-

Table 2: Case study 1: Full data architecture and hyper-parameters for each model and the temporal experiment

Model	Architecture	LR	BS	λ	i
NN	$[2, 128, 256]$	0.0041	2	-	0.2702
BC	$[4, 4]$	0.0184	2	-	2.6972
PP	$[64, 128, 128]$	0.0041	64	-	1.489628
PR	$[8, 256]$	0.0306	2	0.0152	0.62202

den layers. The hyper-parameter search space consists of 300 randomly sampled hyper-parameter vectors. In the combined search each network of the 300 architecture candidates is fitted using the 300 candidate hyper-parameter vectors. Each hyper-parameter vector consists of learning rate, batch size. For the physics embedding and the physics regularisation the hyper-parameter vectors consist additionally of a regularisation factor. The best performing candidate network k is chosen based on the minimum of the index i_k with

$$i_k = \frac{\mathbf{E}\left[\mathcal{L}_{\text{val}}\right]^2 + \sqrt{\mathbf{E}\left[(\mathcal{L}_{\text{val}} - \mathbf{E}\left[\mathcal{L}_{\text{val}}\right])^2\right]}}{2} \quad , \tag{8}$$

where $\mathcal{L}_{\text{val}}$ denotes the validation losses for all cross-validation runs.

The best performing architecture and hyper-parameters are shown for each model in Tab. 6 for the full data experiment and in Tab. 6 for the sparse data experiment.

Table 3: Case study 1: Sparse data architecture and hyper-parameters for each model and the temporal experiment

Model	Architecture	LR	BS	λ	i
NN	$[8, 64, 64, 4]$	0.0224	16	-	0.7872
BC	$[16, 32]$	0.0224	8	-	1.3778
PP	$[2]$	0.0571	64	-	3.9227
PR	$[8, 128]$	0.0041	2	0.0604	1.3862

4.1.2 Bayesian Calibration of the process model

PRELES was re-calibrated using the Bayesian Tools package (see main document). A Markov Chain Monte Carlo simulation using the DREAMzs sampler was used. We ran three chains at 50000 iterations each. The results for case study 1 in the full data scenario are shown in Fig. 10.

4.1.3 Evaluation

The temporal predictions with the full and the sparse data set are shown in Fig. 3 and 4 .

Next, we compare the PM parameters predicted by the physics embedding network in the evaluation with the standalone PM parameters in Tab. 4. We note that we did not calibrate completely the same parameters due to the high sensitivity of the physics embedding network to predicted parameter values. Specifically, during our tests it was apparent that once one PM parameter was estimated outside the range of the PM, PM predictions turned

figures/PsinglesitefitBayesPriorsfull.pdf

Figure 2: Posterior distributions of the re-calibrated PRELES parameters under full data availability.

figures/temporalpredictionsfullcs1.pdf

Figure 3: Case study 1: Temporal predictions over the year 2008 with the full data set.

figures/temporalpredictionssparsecs1.pdf

Figure 4: Case study 1: Temporal predictions over the year 2008 with the sparse data set.

into NAs and the physics embedding could not exit this state during training. Thus, we recommend careful parameter selection.

4.1.4 Inference

The variable importance analysis with the full data set for the embedded network (Embedded$_{raw}$: sensitivity of network predictions, Embedded$_{preles}$: sensitivity of embedded preles predictions) is shown in Fig. 5.

figures/viaembcs1.pdf

Figure 5: Case study 1: Variable importance analysis in June with the full data set for the physics embedding network in the temporal prediction scenario.

The variable importance analysis with the full data set for the bias correction network of **all case studies** is shown in Fig. 6.

Table 4: Case study 1: PM parameters predicted in the physics embedding during evaluation vs. calibrated standalone PM parameters in the full data (top) and sparse data regime (bottom). Mean values are given over all folds with the standard error. An asterisk indicates default parameter values were taken.

Parameter	Physics embedding	PM
p1	412.9147 ± 0.0490	413^*
p5	0.9695 ± 0.0624	0.9686 ± 0.0758
p8	17.1630 ± 0.0237	23.7515 ± 3.6906
p9	0.0256 ± 0.0420	-0.5663 ± 0.0408
p11	0.8937 ± 0.0759	0.7057 ± 0.0641
p14	0.0911 ± 0.0769	3.9940 ± 0.4922
p15	1.0169 ± 0.0732	1.1861 ± 0.0092
p16	-0.0323 ± 0.0399	0.0301 ± 0.0236
p18	0.5550 ± 0.0590	8.9929 ± 0.6212
p19	1.0120 ± 0.0605	1.2^*
p20	0.0795 ± 0.0708	0.33^*
p21	4.9835 ± 0.0763	4.9705^*

Parameter	Physics embedding	PM
p1	413.3656 ± 0.1711	413^*
p5	0.8880 ± 0.0134	0.7635 ± 0.0636
p8	15.3918 ± 0.2146	19.1792 ± 4.8785
p9	0.1690 ± 0.1738	-0.6677 ± 0.0628
p11	1.6106 ± 0.5358	0.8106 ± 0.1463

figures/viares.pdf

Figure 6: Variable importance analysis in June with the full data set for the bias correction network of **all case studies** (see columns).

4.2 Case study 2: spatial multi-site prediction

4.2.1 Network architecture and hyperparameter search

We run a combined random grid search to specify network architectures and hyperparameters, identical to case study 1.

4.2.2 Bayesian Calibration of the process model

4.2.3 Evaluation

The spatial predictions for the full evaluation year 2008 given the full and sparse data set are shown in Fig. 7 and 8.

figures/temporalpredictionsfullcs2.pdf

Figure 7: Case study 2: Spatial predictions over the year 2008 with the full data set.

figures/temporalpredictionssparsecs2.pdf

Figure 8: Case study 2: Spatial predictions over the year 2008 with the sparse data set.

Next, we compare the PM parameters predicted by the physics embedding network in the evaluation with the standalone PM parameters in Tab. 7.

Table 5: Case study 2-3: Full data architecture and hyper-parameters for each model and the temporal experiment

Model	Architecture	LR	BS	λ	i
NN	$[2, 16]$	0.0041	2	-	4.6764
BC	$[2, 8, 4, 16]$	0.0082	32	-	10.4112
PP	$[2, 8]$	0.0306	64	-	5.8998
PR	$[2, 16, 16]$	0.0061	4	0.0051	6.3038

Table 6: Case study 2-3: Sparse data architecture and hyper-parameters for each model and the temporal experiment

Model	Architecture	LR	BS	λ	i
NN	$[2, 128, 32, 8]$	0.002	8	-	5.1329
BC	$[2, 16]$	0.0796	64	-	16.2473
PP	$[2]$	0.0082	32	-	5.4535
PR	$[2, 128, 4, 32]$	0.0061	32	0.0704	5.8726

Table 7: Case study 2: PM parameters predicted in the physics embedding during evaluation vs. calibrated standalone PM parameters in the full data (top) and sparse data regime (bottom). Mean values are given over all folds with the standard error. An asterisk indicates default parameter values were taken.

Parameter	Physics embedding	PM
p1	412.9727 ± 0.0995	413^*
p5	1.1879 ± 0.0513	0.8834 ± 0.1586
p8	17.1789 ± 0.0999	21.9373 ± 4.3287
p9	-0.0530 ± 0.1134	-0.4260 ± 0.1451
p11	0.8986 ± 0.0762	0.7673 ± 0.0545
p14	0.0382 ± 0.1121	0.8742 ± 0.6853
p15	1.1055 ± 0.1056	0.3199 ± 0.2917
p16	0.0624 ± 0.0510	0.0765 ± 0.0443
p18	0.5265 ± 0.0631	1.5372 ± 1.5135
p19	1.1724 ± 0.0574	1.2^*
p20	0.0453 ± 0.1581	0.33^*
p21	4.9367 ± 0.1003	4.9705^*

Parameter	Physics embedding	PM
p1	412.8733 ± 0.0639	413^*
p5	0.9528 ± 0.0405	1.1884 ± 0.1427
p8	16.9842 ± 0.0869	19.9236 ± 2.7435
p9	0.02389 ± 0.0662	-0.5355 ± 0.1429
p11	0.8094 ± 0.0574	0.5721 ± 0.1418

4.2.4 Inference

The variable importance analysis with the full data set for the physics embedding network (Embedded$_{raw}$: sensitivity of network predictions, Embedded$_{preles}$: sensitivity of embedded preles predictions) is shown in Fig. 9.

figures/viaembcs2.pdf

Figure 9: Case study 2: Variable importance analysis in June with the full data set for the physics embedding network in the spatial prediction scenario.

4.3 Case study 3: spatio-temporal prediction

4.3.1 Network architecture and hyperparameter search

For simplicity, we use the results of the architecture and hyperparameter search of case study 2 to define model parameters in this case study.

4.3.2 Bayesian Calibration of the process model

PRELES was re-calibrated using the Bayesian Tools package (see main document). A Markov Chain Monte Carlo simulation using the DREAMzs sampler was used. We ran three chains at 50000 iterations each. The results for case study 3 in the full data scenario are shown in Fig. 10.

figures/PmultisitefitBayesPriorsexp3full.pdf

Figure 10: Posterior distributions for case study 3 of the spatio-temporal re-calibrated PRELES parameters under full data availability.

4.3.3 Evaluation

The spatio-temporal predictions for the full evaluation year 2008 with the full and sparse data set are shown in Fig. 11 and 12.

figures/temporalpredictionsfullcs3.pdf

Figure 11: Case study 3: Spatio-temporal predictions for the year 2008 with the full data set.

figures/temporalpredictionssparsecs3.pdf

Figure 12: Case study 3: Spatio-temporal predictions for the year 2008 with the full data set.

Next, we compare the PM parameters predicted by the physics embedding network in the evaluation with the standalone PM parameters in Tab. 8.

4.3.4 Inference

The variable importance analysis with the full data set for the physics embedding network (Embedded$_{raw}$: sensitivity of network predictions, Embedded$_{preles}$: sensitivity of embedded preles predictions) is shown in Fig. 13.

Table 8: Case study 3: PM parameters predicted in the physics embedding during evaluation vs. calibrated standalone PM parameters in the full data (top) and sparse data regime (bottom). Mean values are given over all folds with the standard error. An asterisk indicates default parameter values were taken.

Parameter	Physics embedding	PM
p1	412.9717 ± 0.0651	413^*
p5	0.9227 ± 0.1217	0.9240 ± 0.1395
p8	16.9626 ± 0.1394	21.9664 ± 4.9185
p9	0.0994 ± 0.0433	-0.5393 ± 0.0737
p11	0.9101 ± 0.0574	0.7717 ± 0.0710
p14	0.0722 ± 0.0645	3.3053 ± 1.7754
p15	1.0419 ± 0.0902	0.6728 ± 0.2993
p16	-0.0066 ± 0.0662	0.0410 ± 0.0335
p18	0.5269 ± 0.0379	4.4528 ± 2.5584
p19	0.8905 ± 0.1166	1.2^*
p20	-0.0113 ± 0.0450	0.33^*
p21	4.9852 ± 0.0545	4.9705^*

Parameter	Physics embedding	PM
p1	413.1141 ± 0.0251	413^*
p5	1.1132 ± 0.0634	1.3742 ± 0.2845
p8	17.0775 ± 0.0915	19.8289 ± 4.2376
p9	-0.0363 ± 0.0218	-0.7323 ± 0.0776
p11	0.8814 ± 0.0518	0.6183 ± 0.1606

figures/viaembcs3.pdf

Figure 13: Case study 3: Variable importance analysis in June with the full data set for the physics embedding network in the spatio-temporal prediction scenario.
