# Supplementary material for: Process‐Informed Neural Networks: A Hybrid Modelling Approach to Improve Predictive Performance and Inference of Neural Networks in Ecology and Beyond
Source: Ecol Lett. 2024 Dec 3;27(11):e70012. doi: 10.1111/ele.70012 (PMC11613309; doi:10.1111/ele.70012)
Supplement: Supplementary file 1 — Data S1. [file ELE-27-0-s001.zip › sim_results_all.pdf]

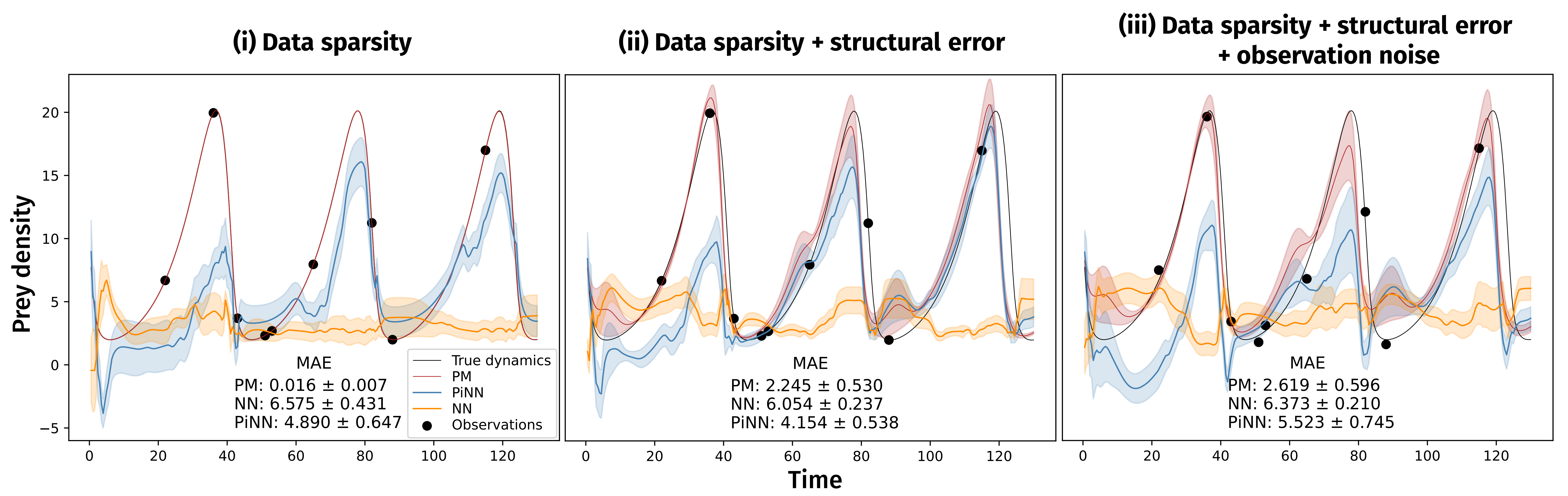

(i) Data sparsity
(ii) Data sparsity + structural error
(iii) Data sparsity + structural error + observation noise
Prey density
Time
True dynamics
PM
PiNN
NN
Observations
MAE
PM: 0.016 ± 0.007
NN: 6.575 ± 0.431
PiNN: 4.890 ± 0.647
MAE
PM: 2.245 ± 0.530
NN: 6.054 ± 0.237
PiNN: 4.154 ± 0.538
MAE
PM: 2.619 ± 0.596
NN: 6.373 ± 0.210
PiNN: 5.523 ± 0.745
