# Supplementary material for: Process‐Informed Neural Networks: A Hybrid Modelling Approach to Improve Predictive Performance and Inference of Neural Networks in Ecology and Beyond
Source: Ecol Lett. 2024 Dec 3;27(11):e70012. doi: 10.1111/ele.70012 (PMC11613309; doi:10.1111/ele.70012)
Supplement: Supplementary file 1 — Data S1. [file ELE-27-0-s001.zip › temporalpredictions_full_cs2.pdf]

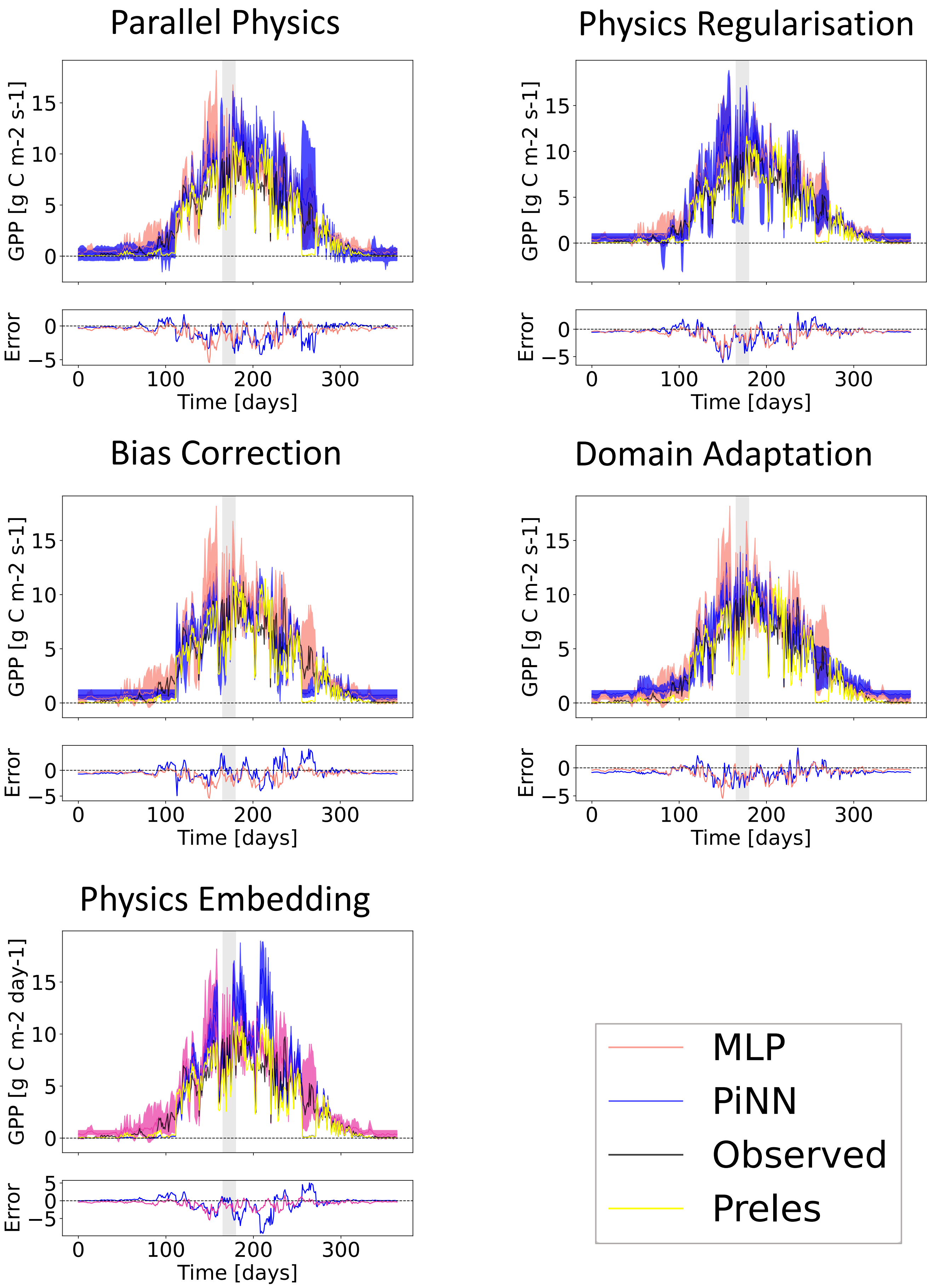
Parallel Physics
Physics Regularisation
Bias Correction
Domain Adaptation
Physics Embedding
GPP [g C m-2 s-1]
GPP [g C m-2 day-1]
Error
Time [days]
0
5
10
15
−5
100
200
300
MLP
PiNN
Observed
Preles
