# Supplementary figures and images for: Process‐Informed Neural Networks: A Hybrid Modelling Approach to Improve Predictive Performance and Inference of Neural Networks in Ecology and Beyond
Source: Ecol Lett. 2024 Dec 3;27(11):e70012. doi: 10.1111/ele.70012 (PMC11613309; doi:10.1111/ele.70012)

## A. Data usage

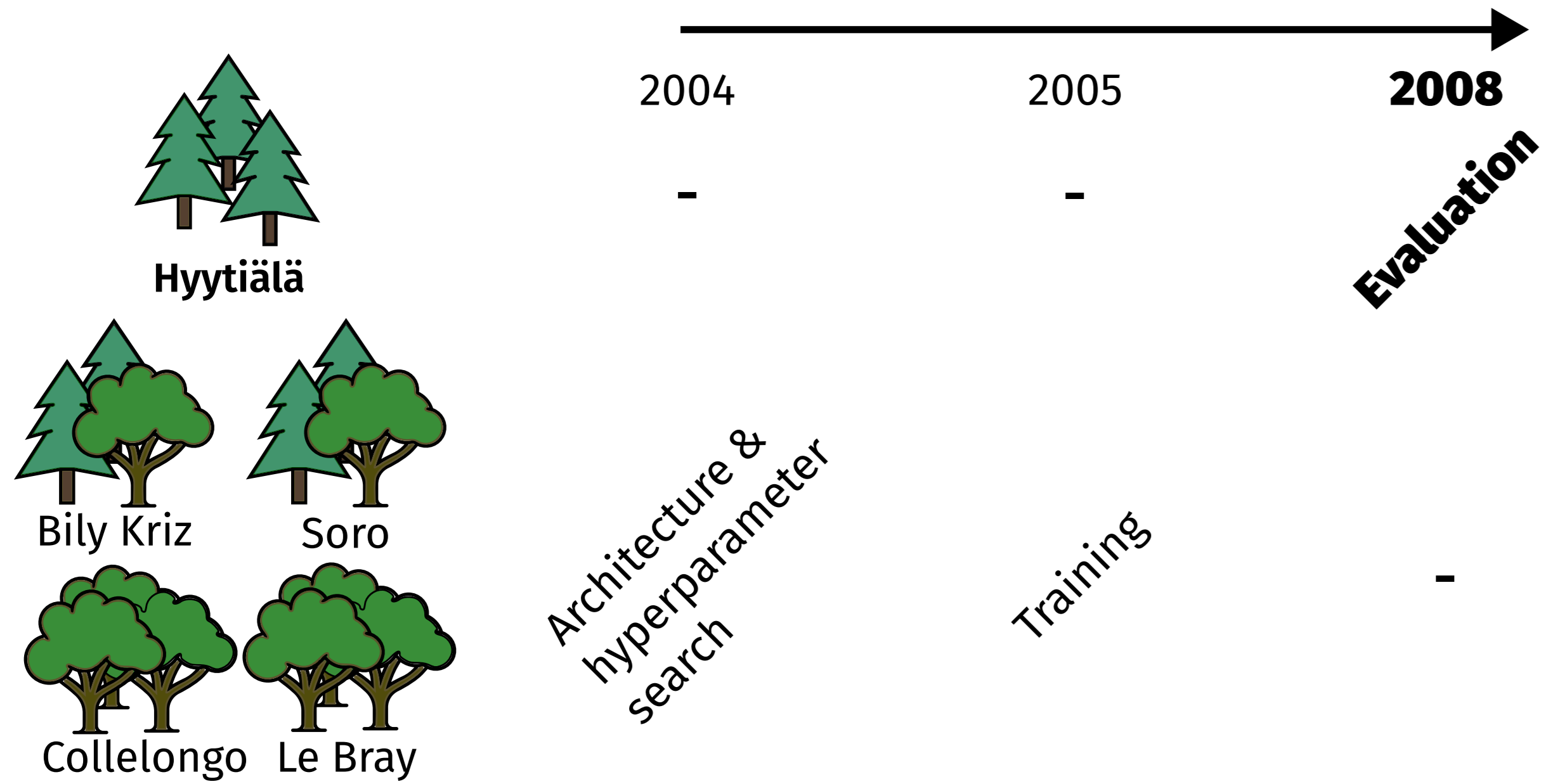

## C. Inference

### Variable importance in June

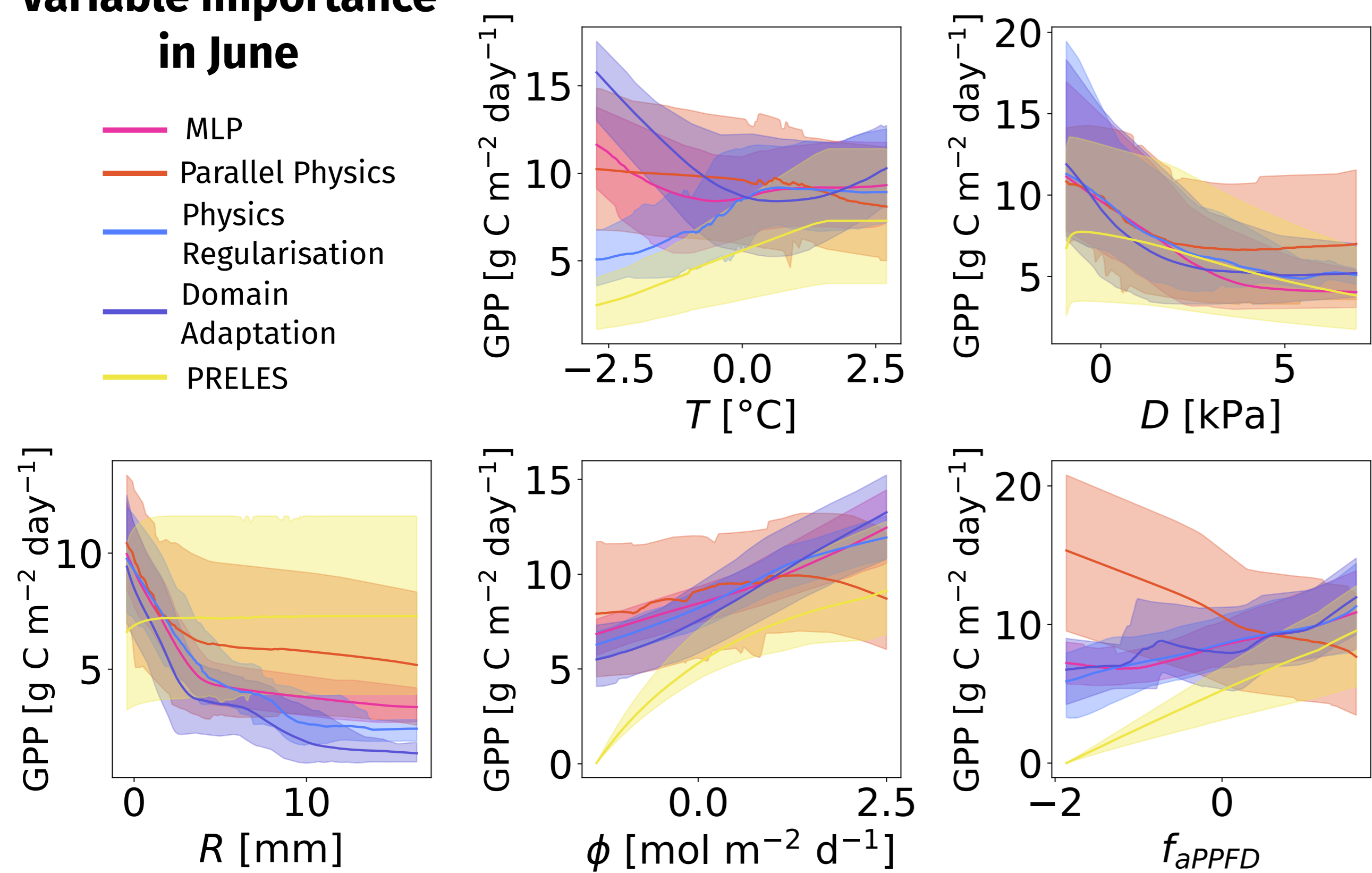

## B. Evaluation

1.

Accuracy

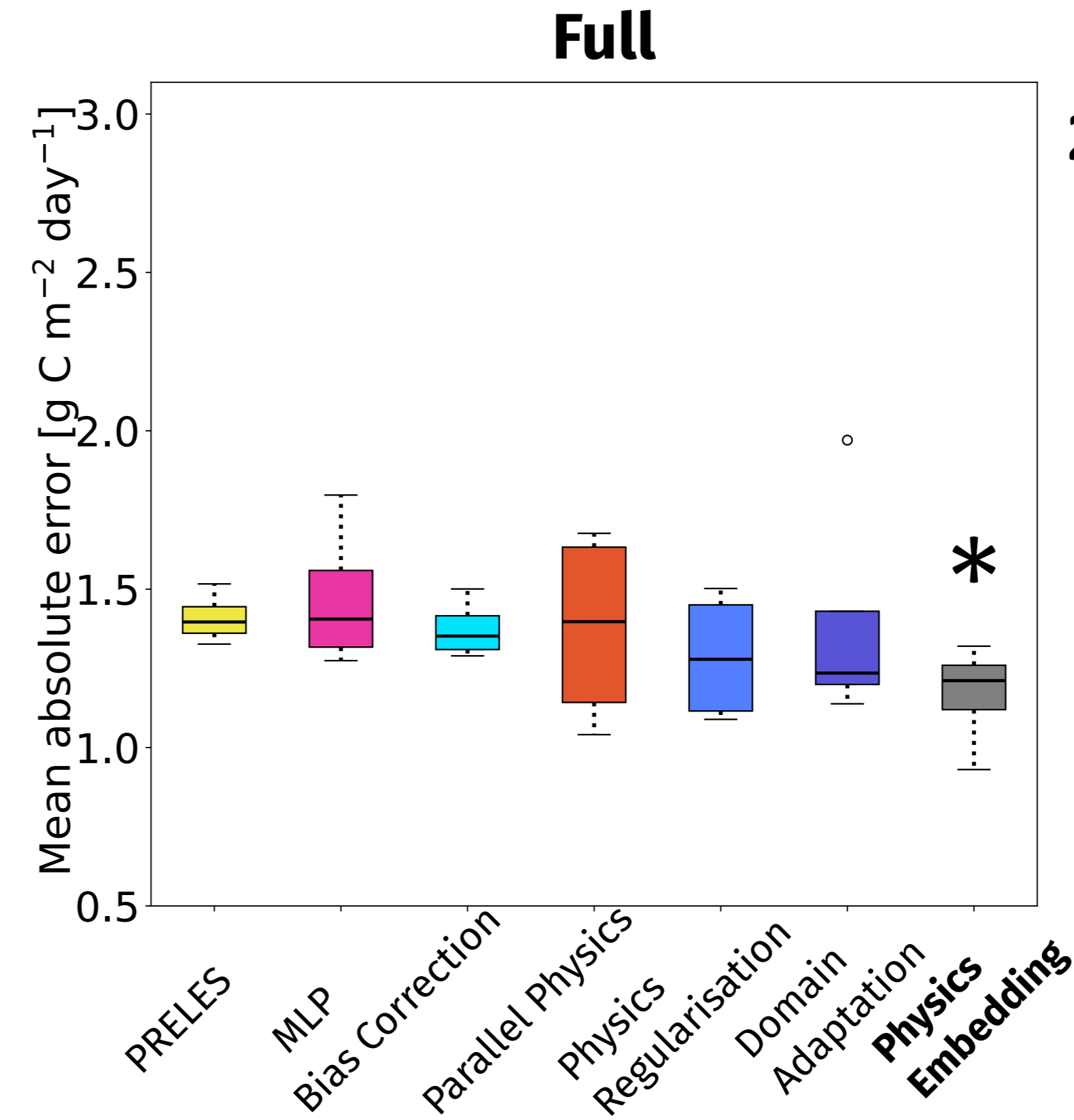

2.

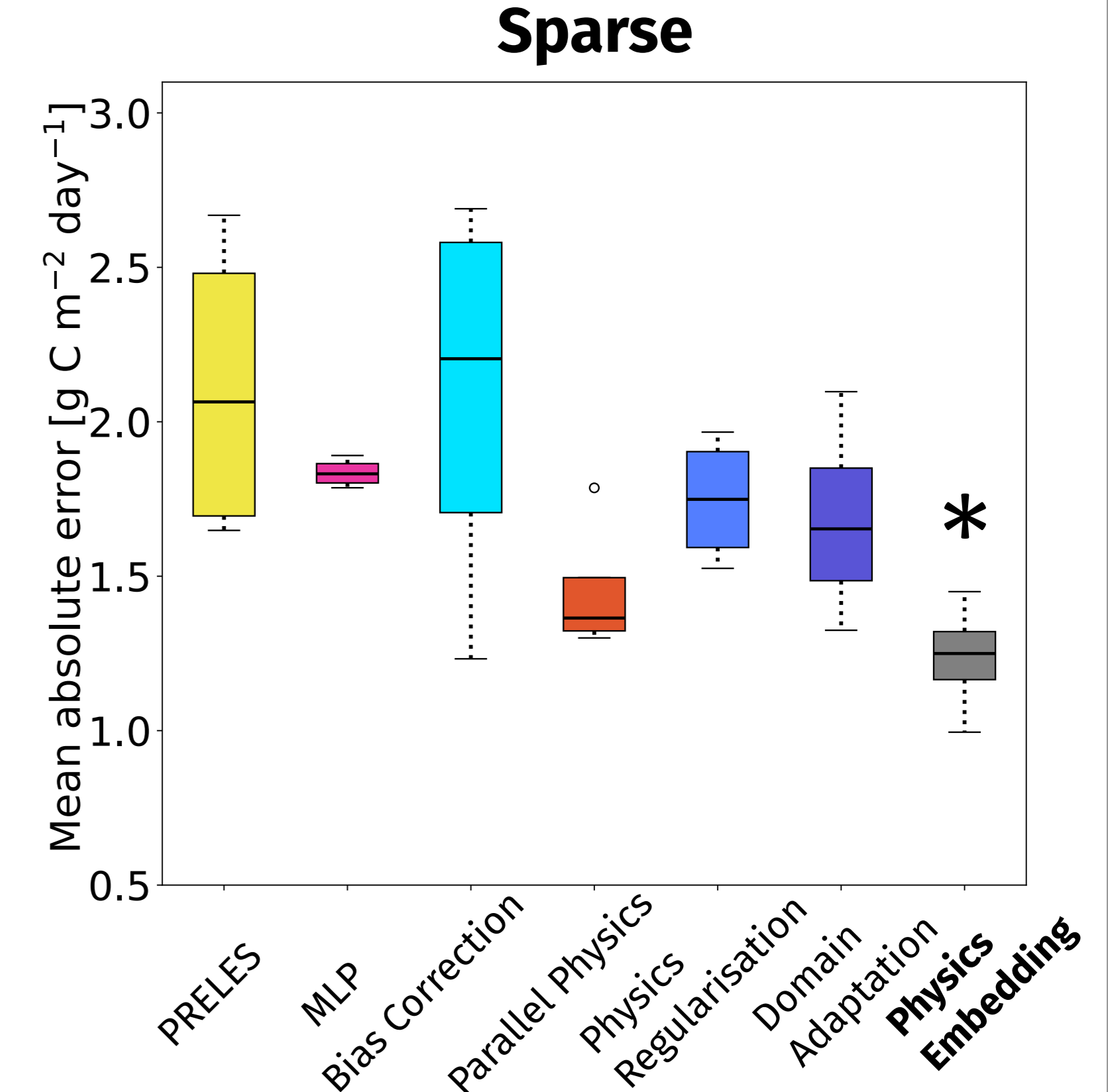

3.

Association

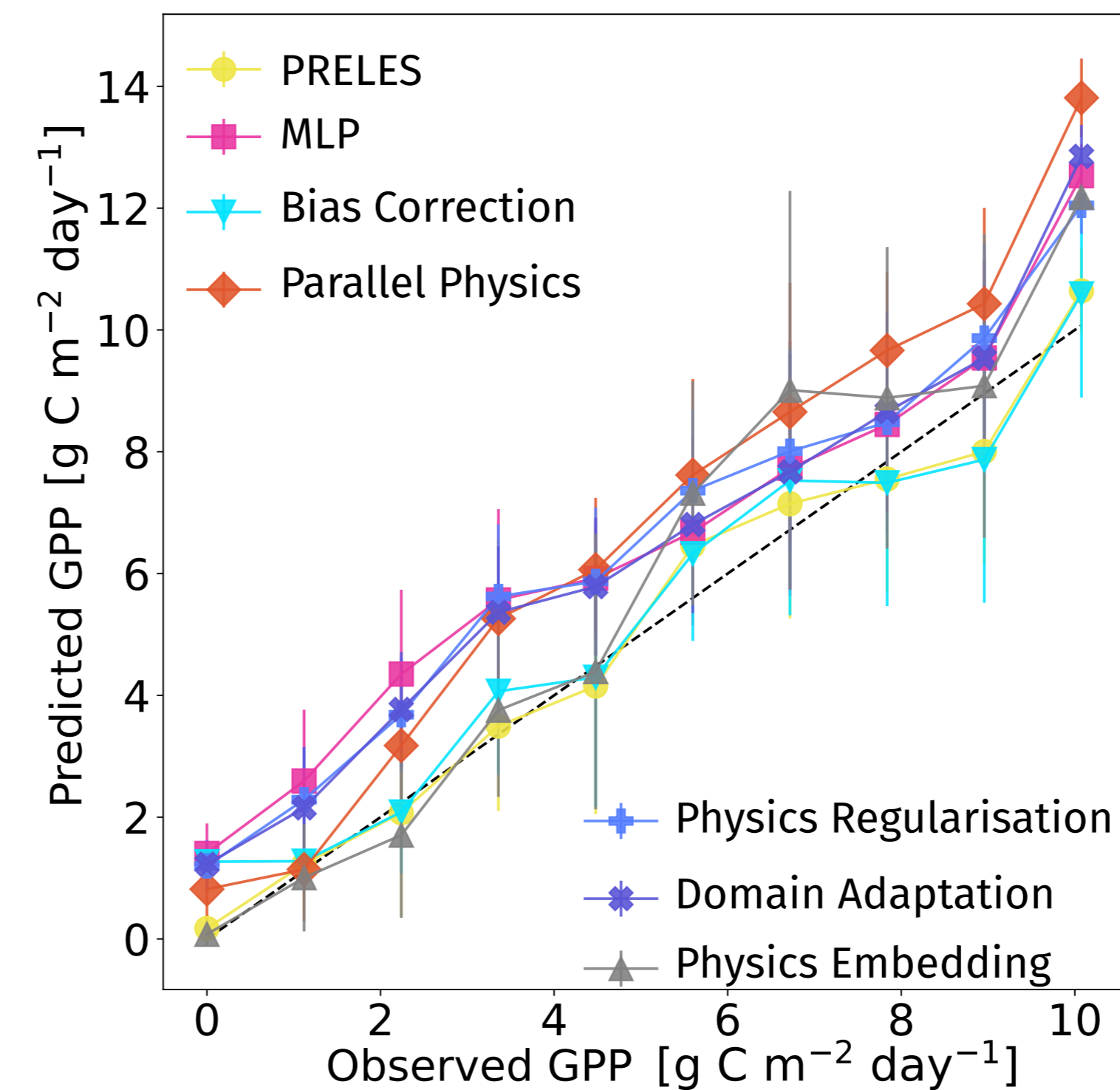

4.

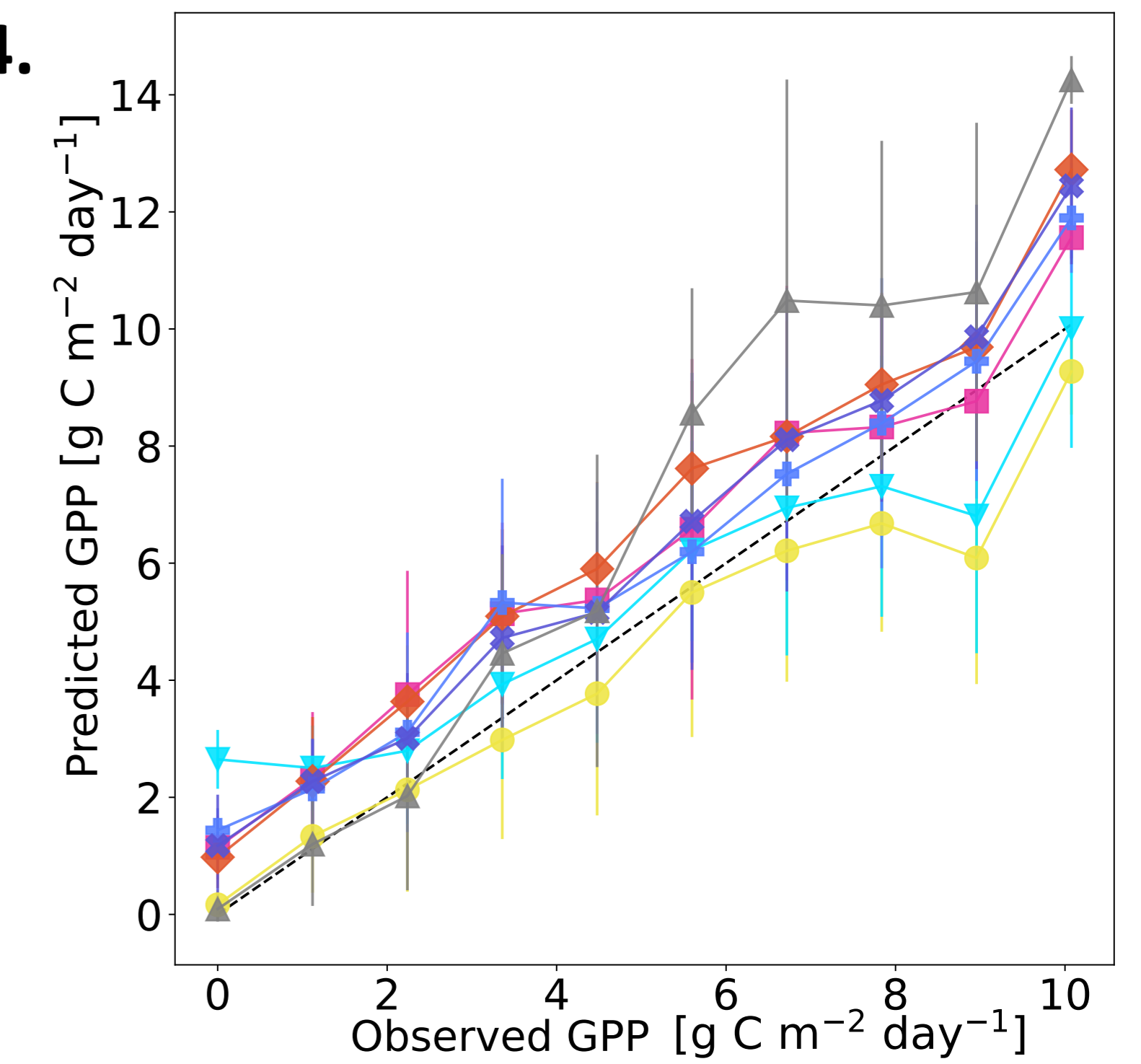

Supplement: Supplementary file 1 — Data S1. [file ELE-27-0-s001.zip › CaseStudy3.pdf]

# Expected performance sweet spots

**Data availability**

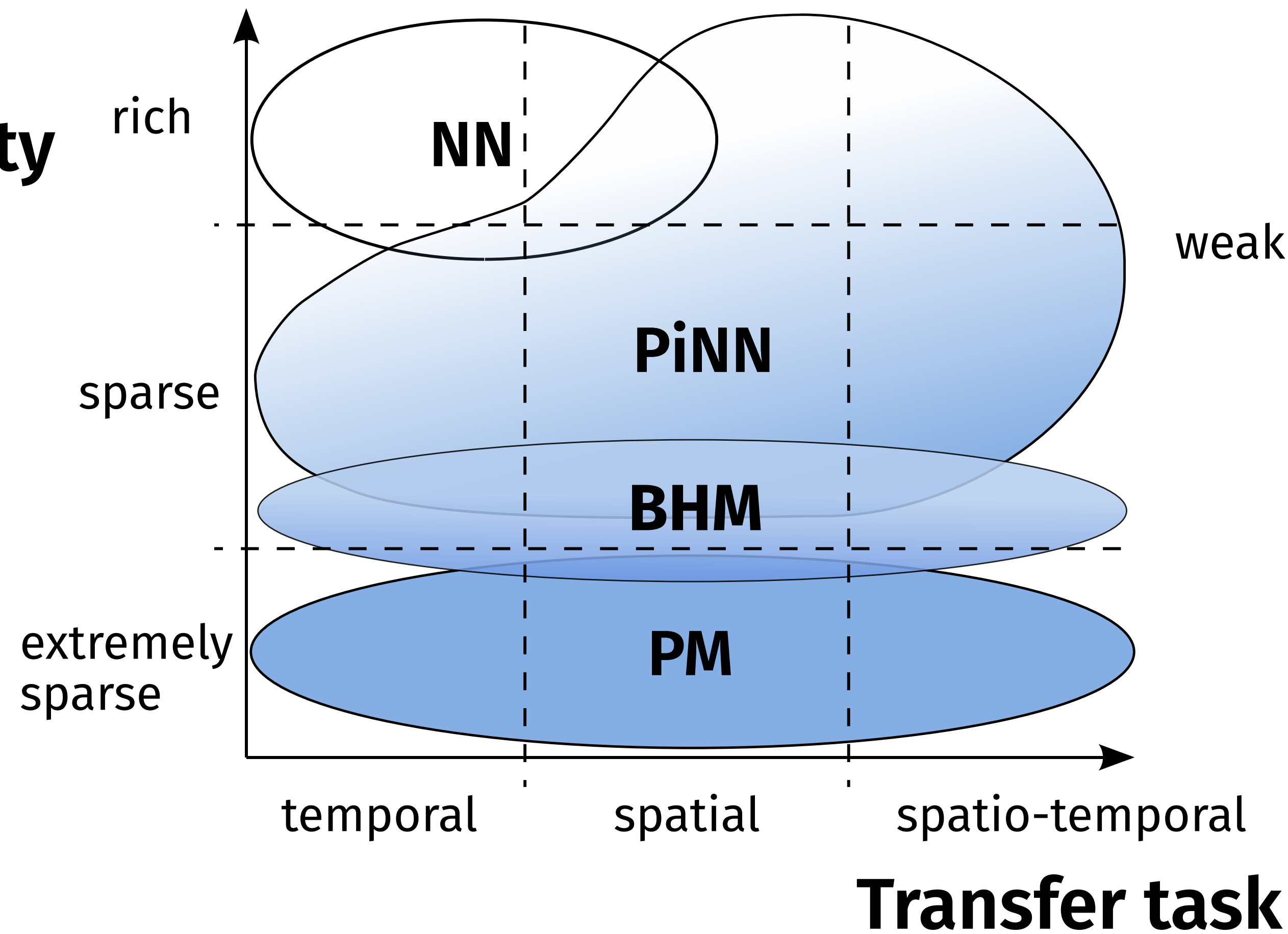

## Theory constraint of PiNNs

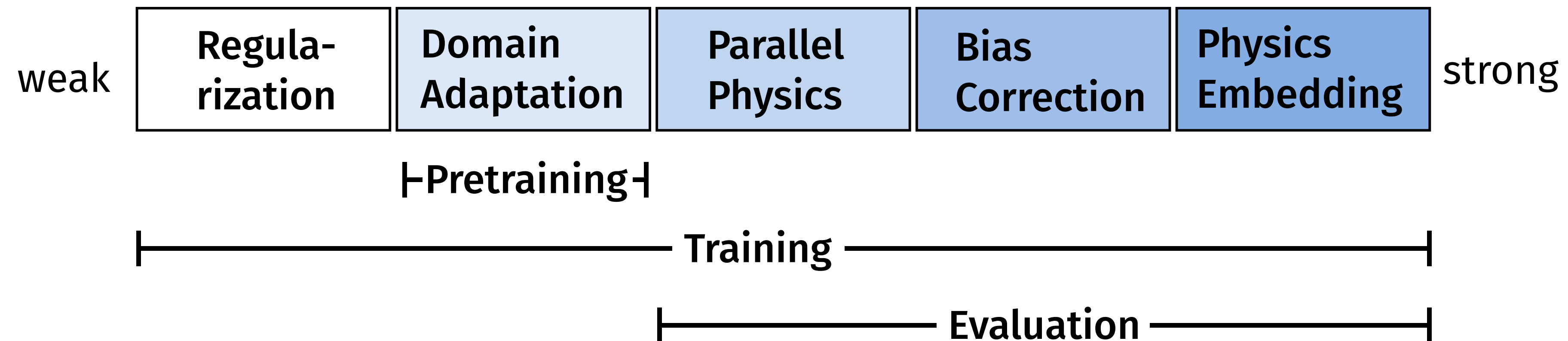

Supplement: Supplementary file 1 — Data S1. [file ELE-27-0-s001.zip › data_theory_dichotomy.pdf]

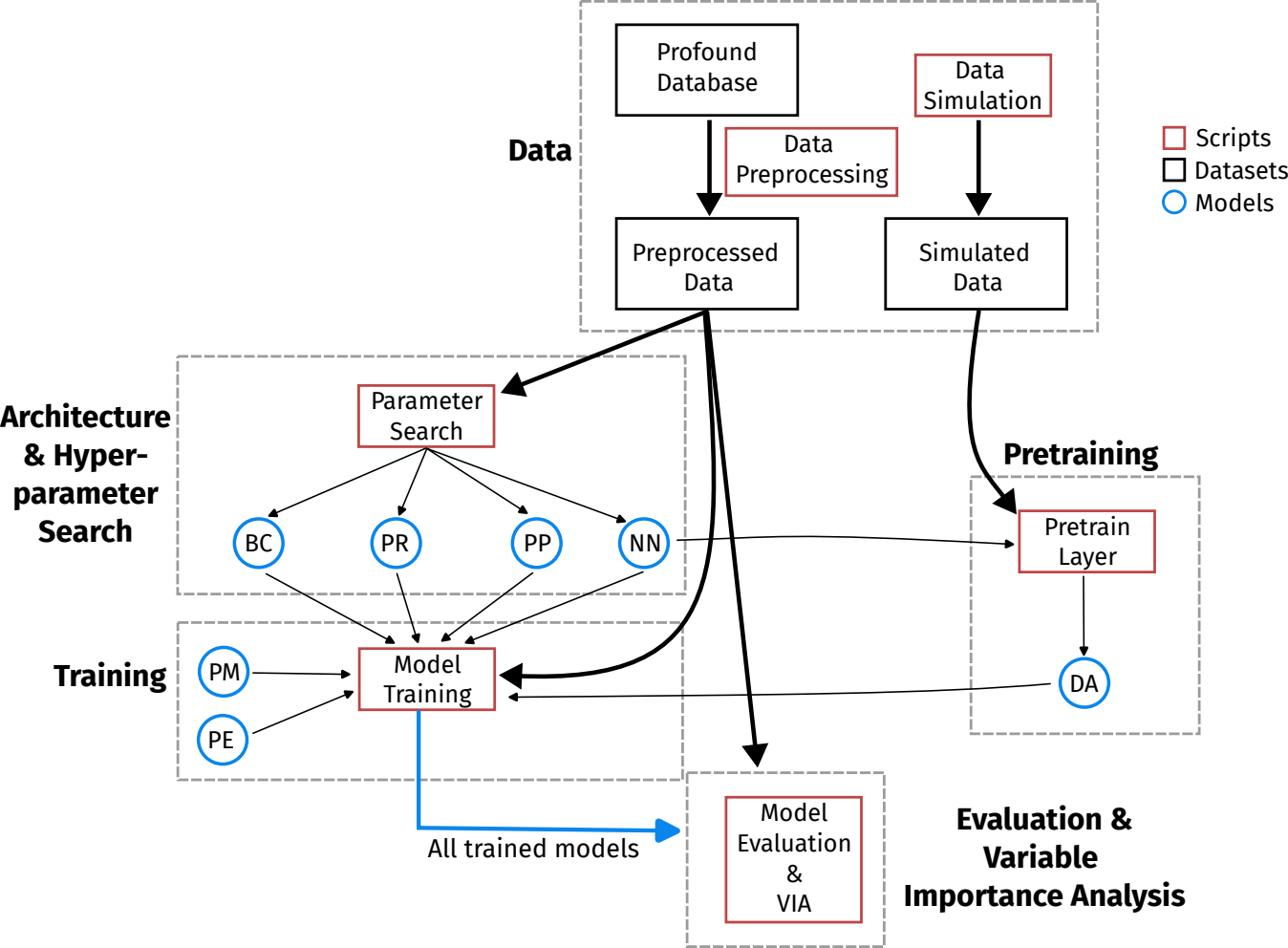

Supplement: Supplementary file 1 — Data S1. [file ELE-27-0-s001.zip › pipeline.pdf]

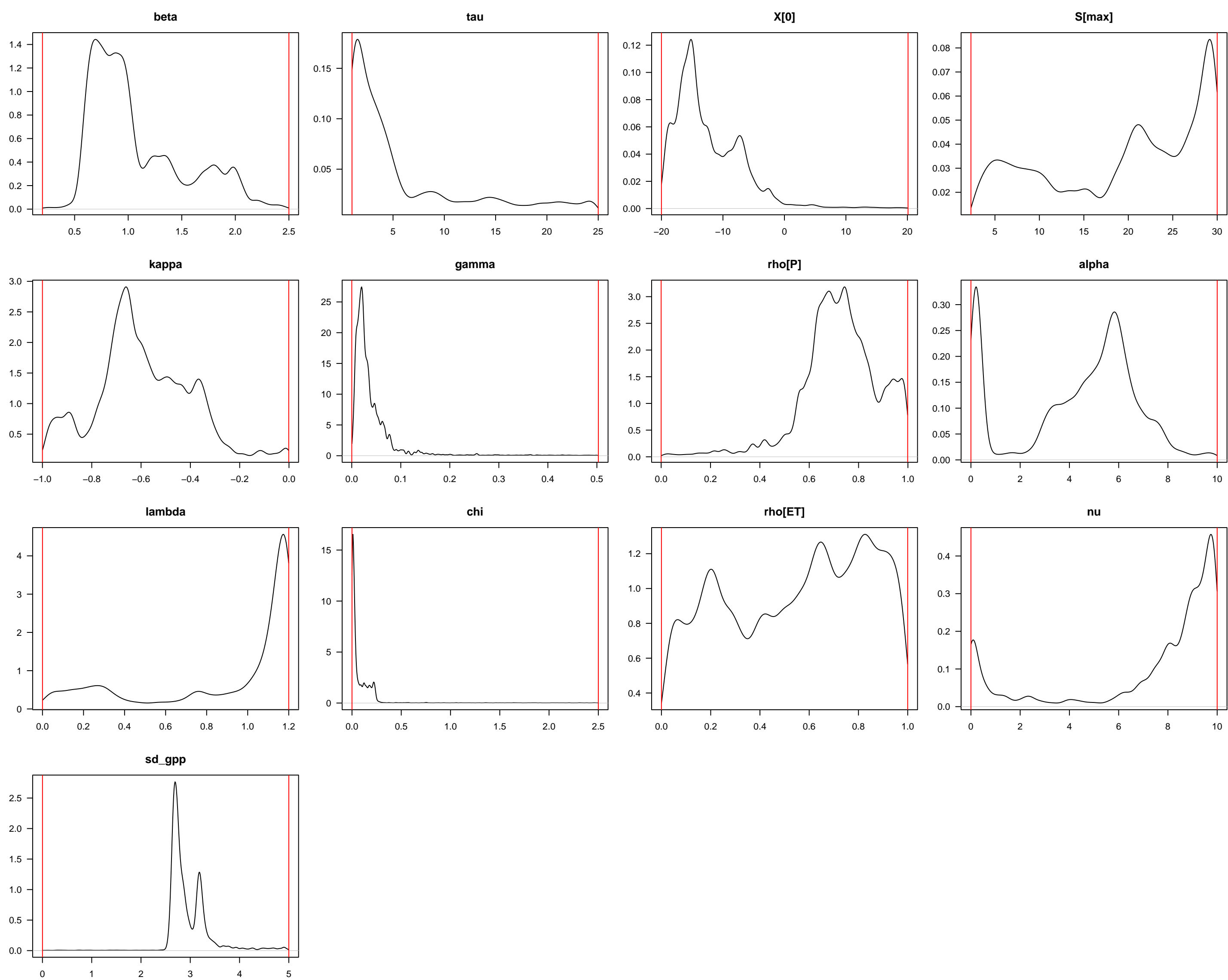

Supplement: Supplementary file 1 — Data S1. [file ELE-27-0-s001.zip › Pmultisite_fit_BayesPriors_exp3_full.pdf]

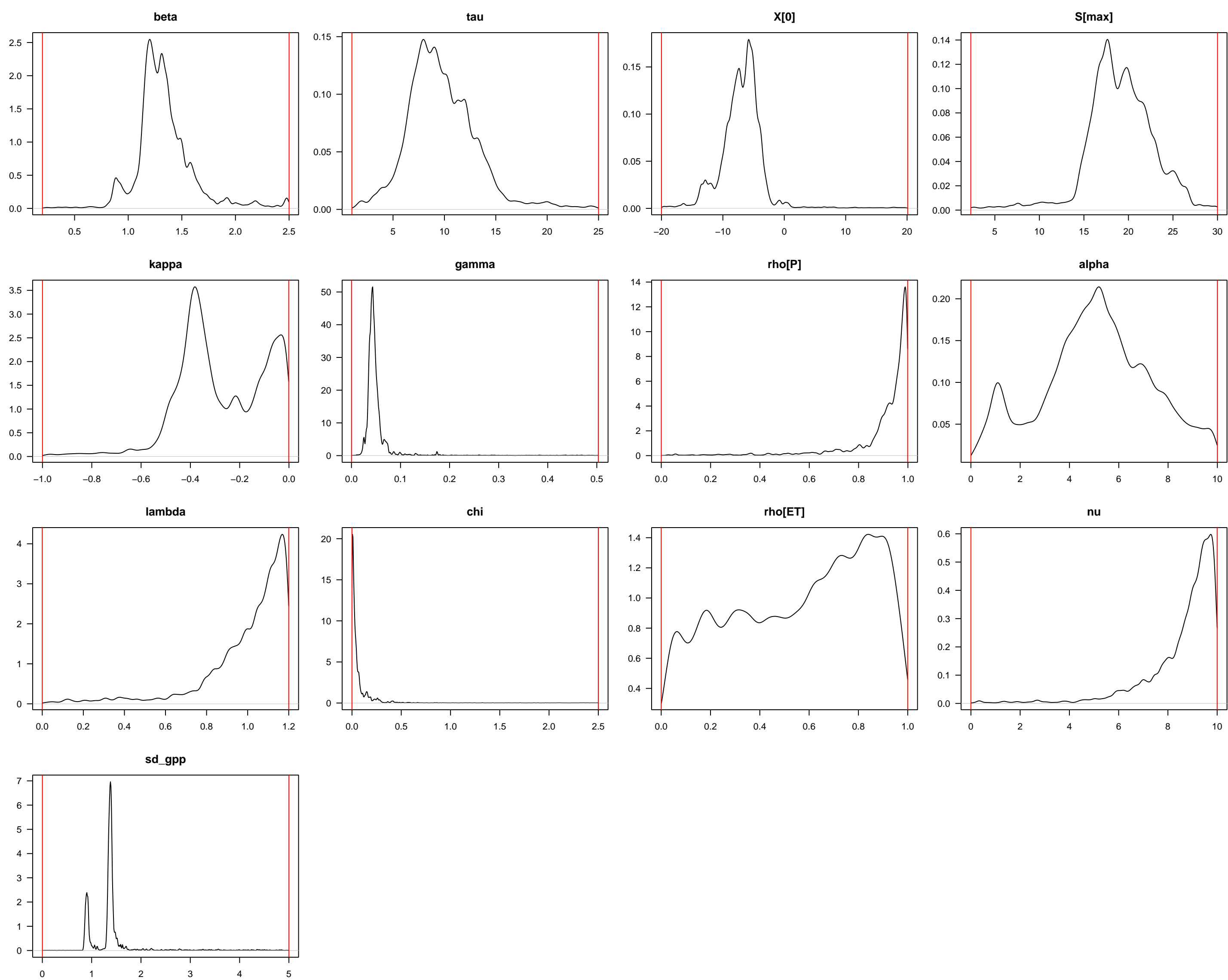

Supplement: Supplementary file 1 — Data S1. [file ELE-27-0-s001.zip › Psinglesite_fit_BayesPriors_full.pdf]

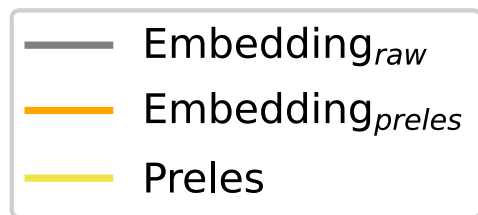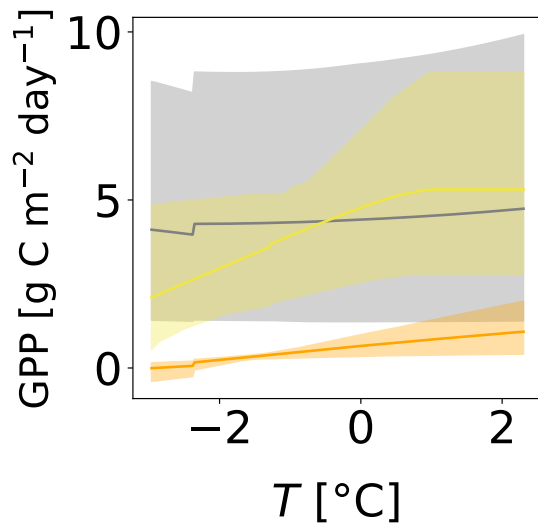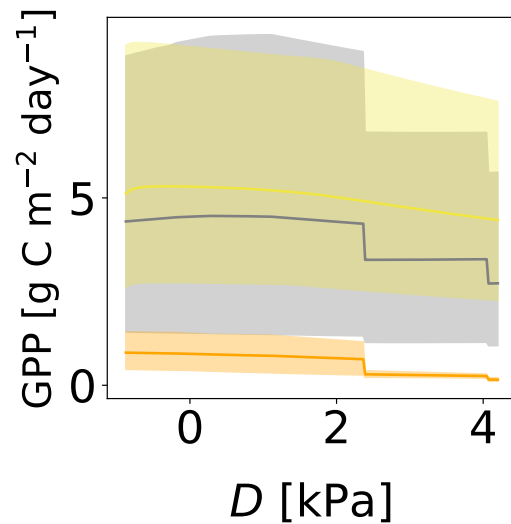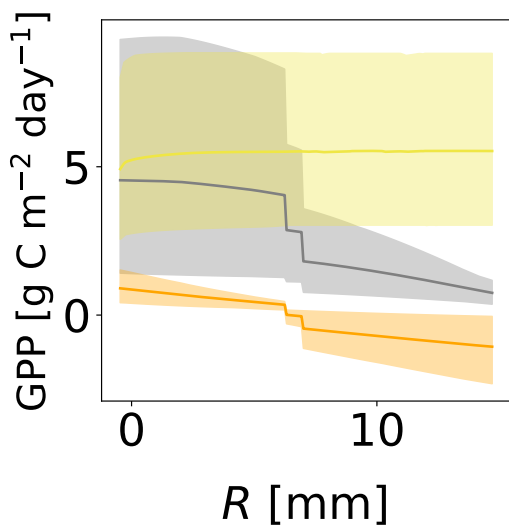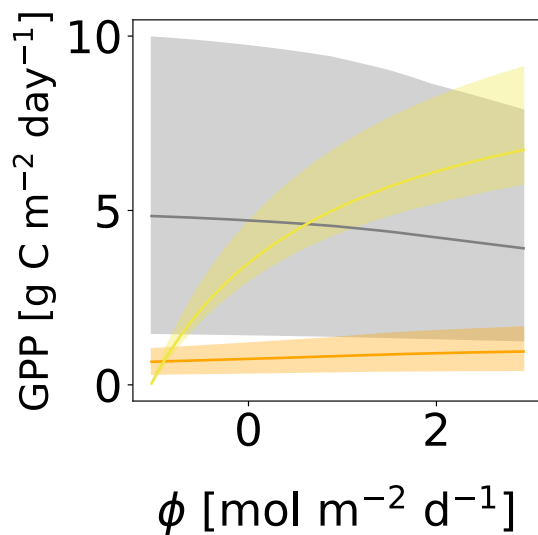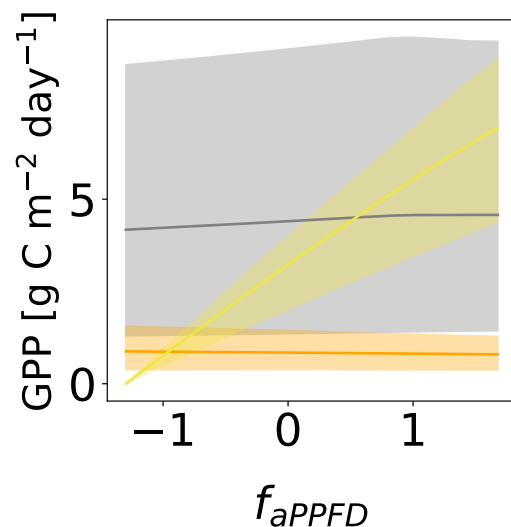

Supplement: Supplementary file 1 — Data S1. [file ELE-27-0-s001.zip › via_emb_cs1.pdf]

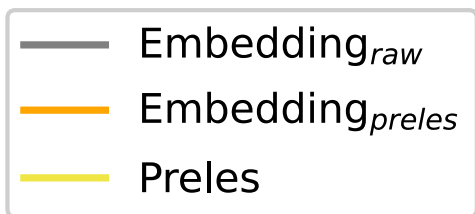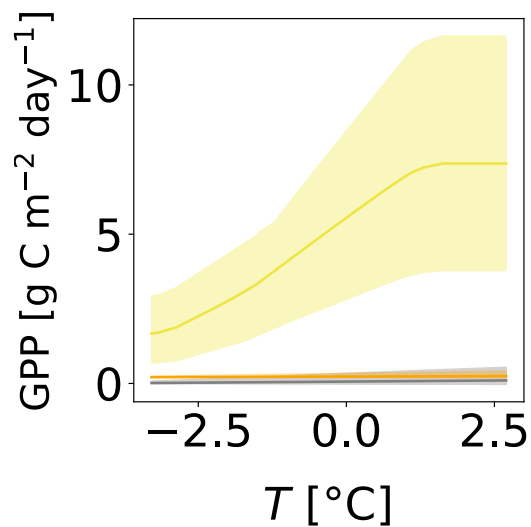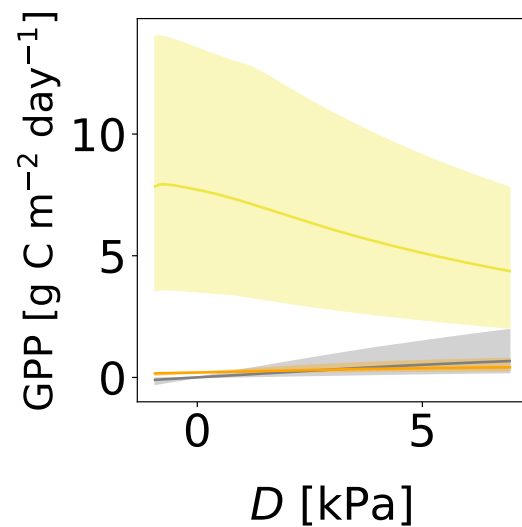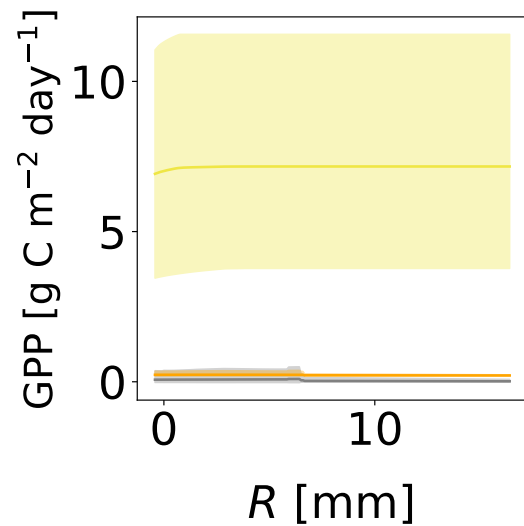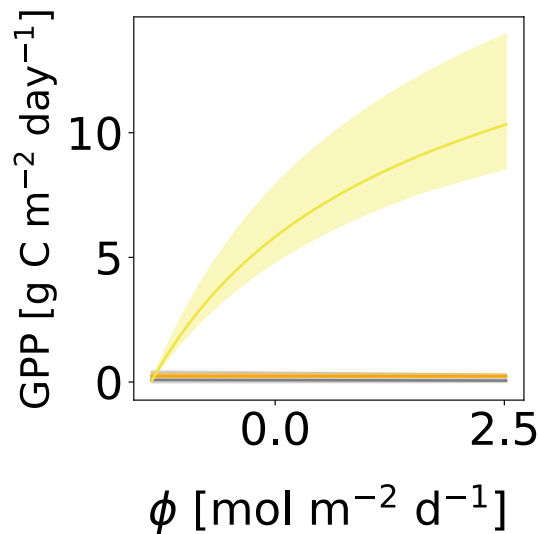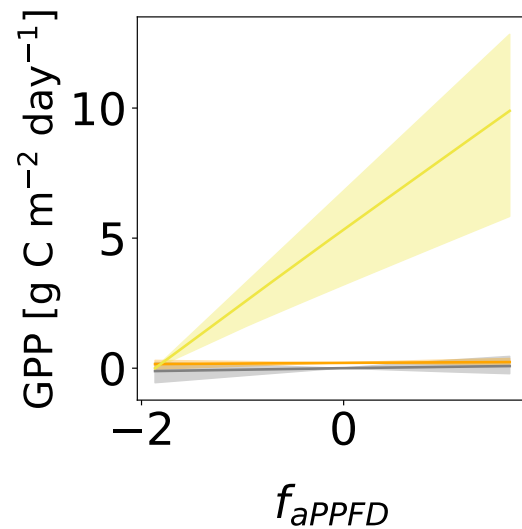

Supplement: Supplementary file 1 — Data S1. [file ELE-27-0-s001.zip › via_emb_cs2.pdf]

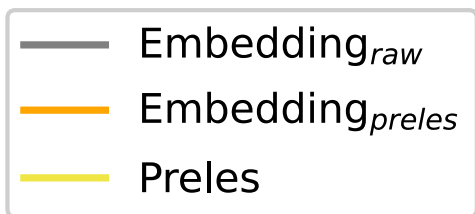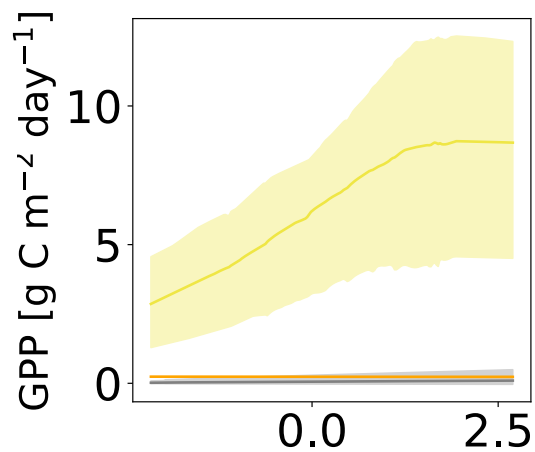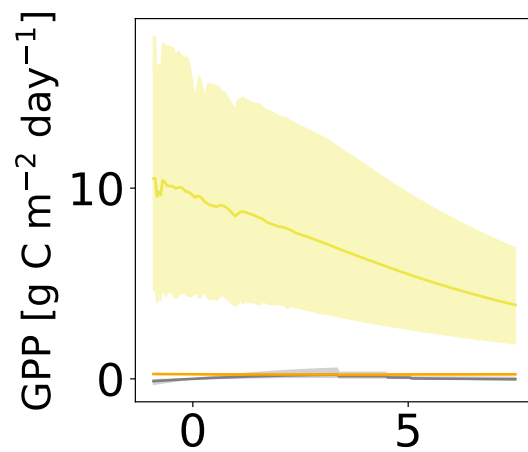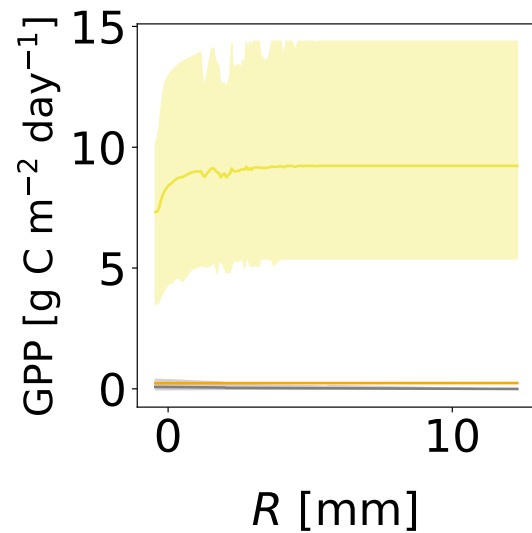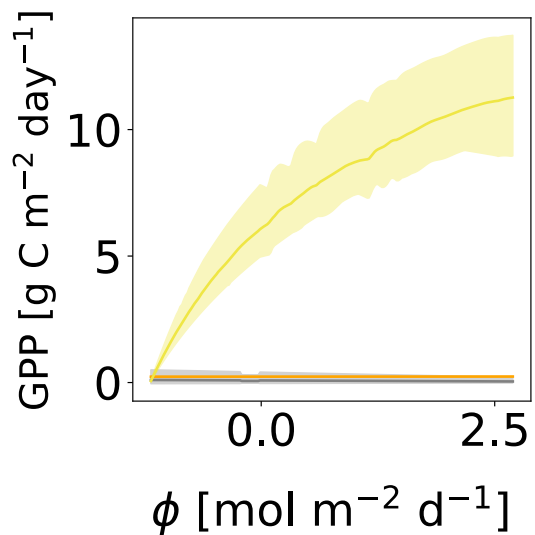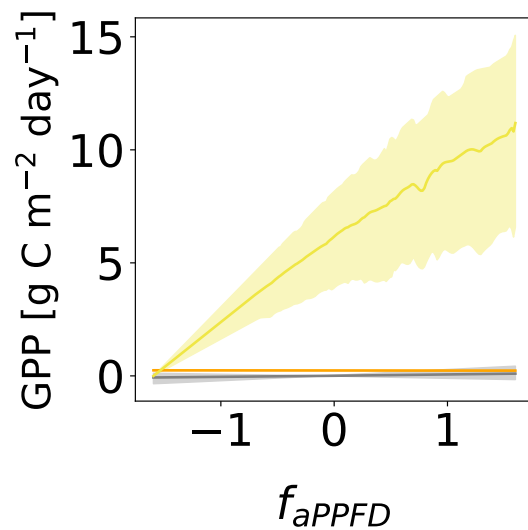

Supplement: Supplementary file 1 — Data S1. [file ELE-27-0-s001.zip › via_emb_cs3.pdf]

Predicted GPP [ $\text{g C m}^{-2} \text{ day}^{-1}$ ]

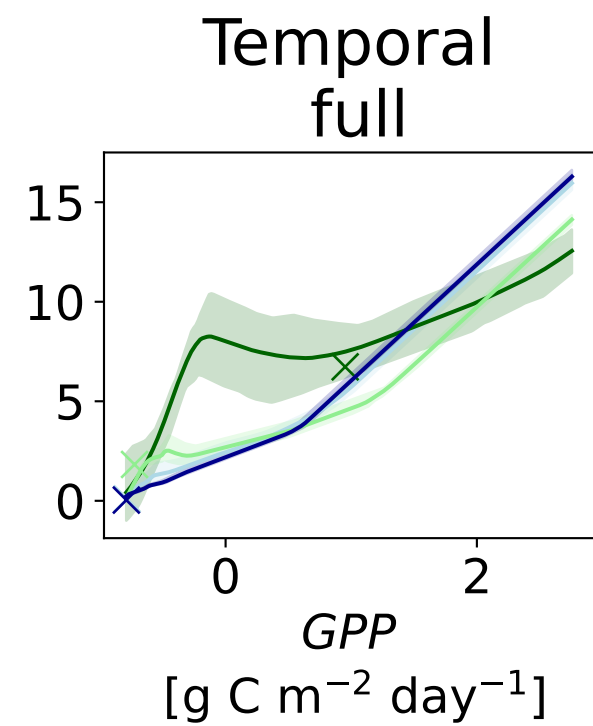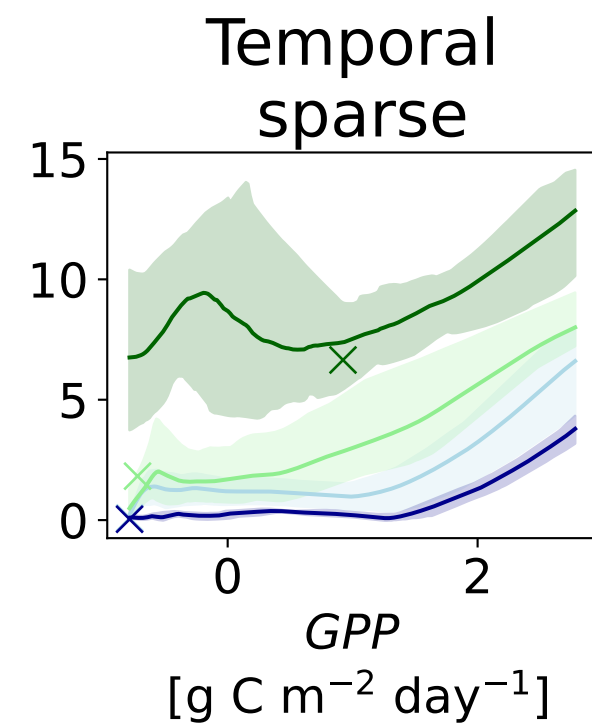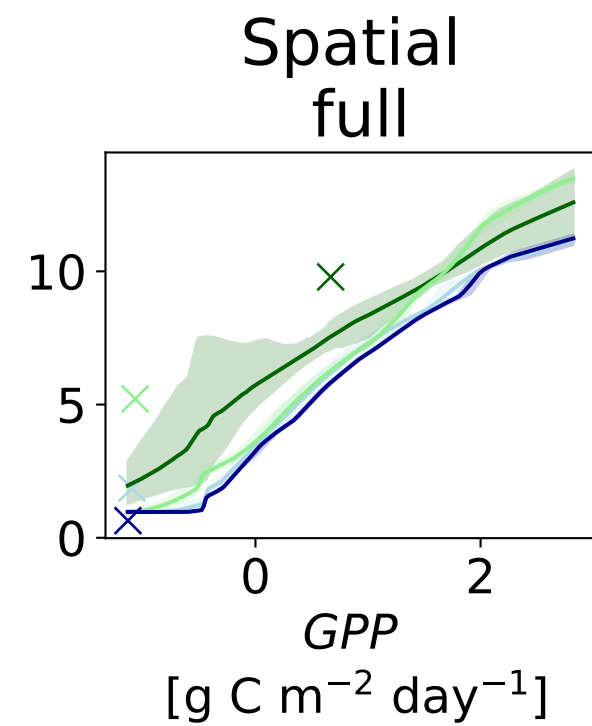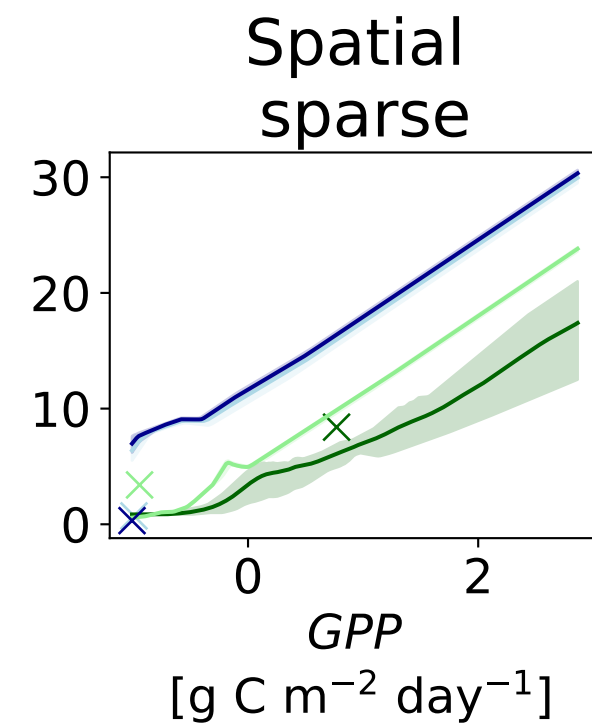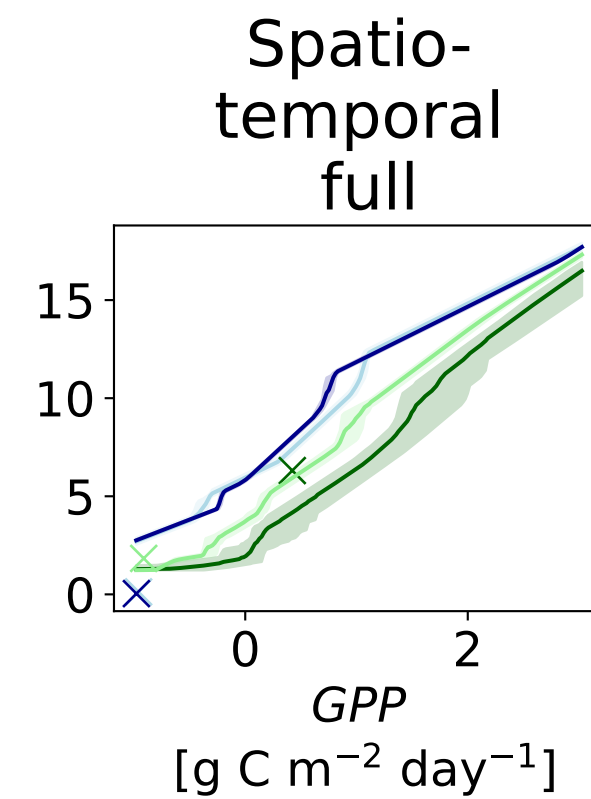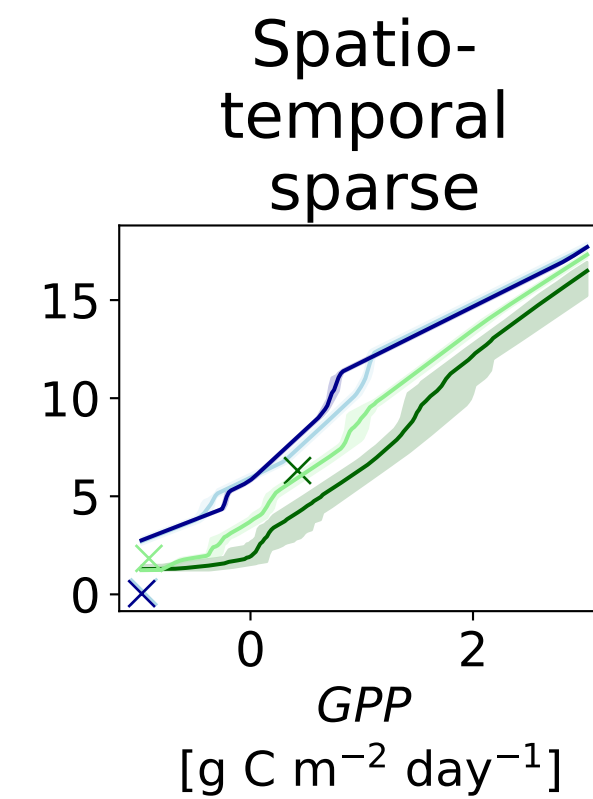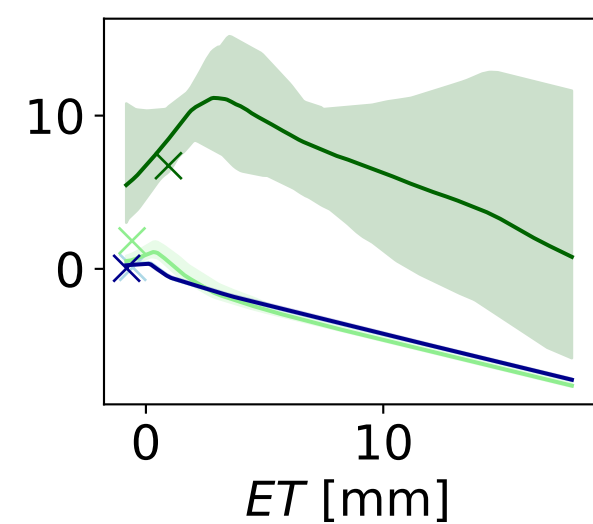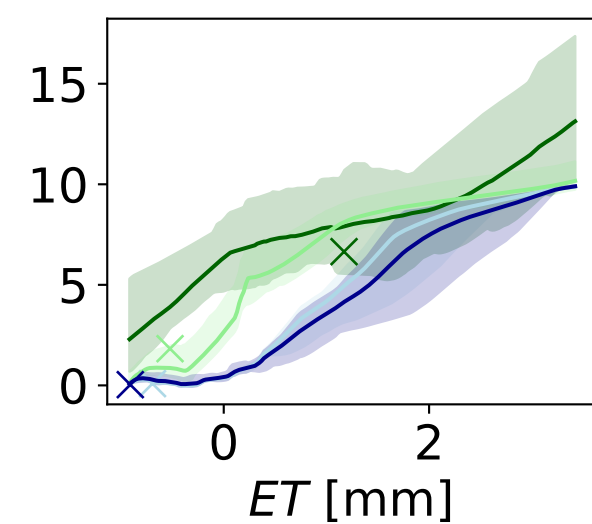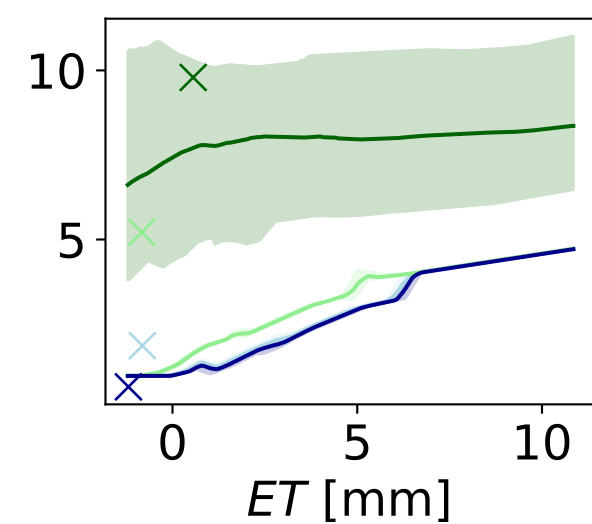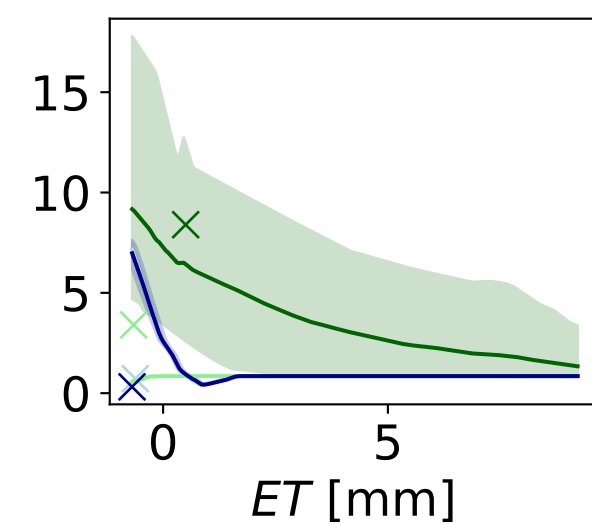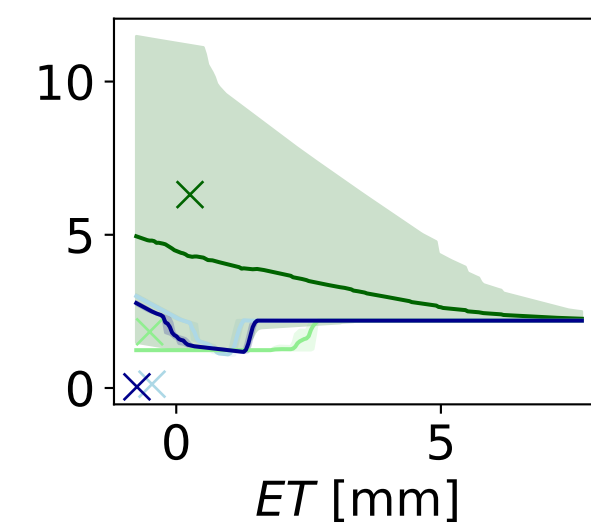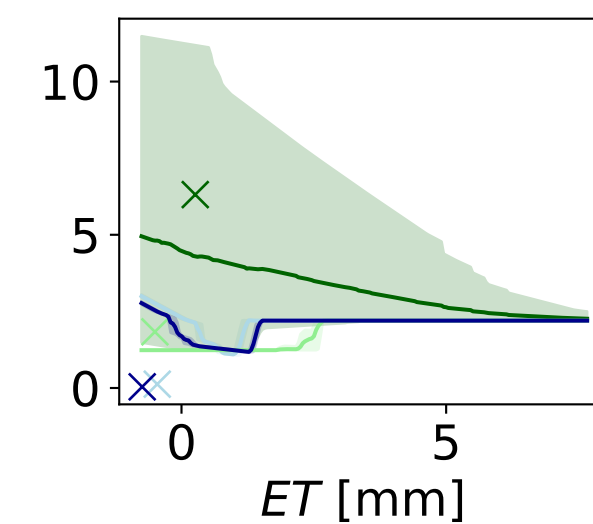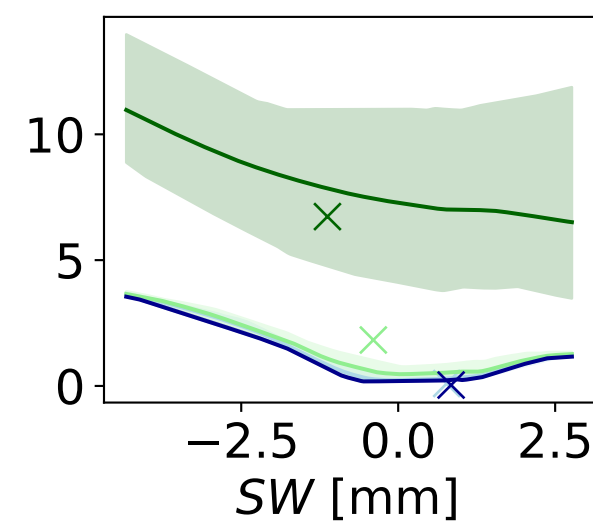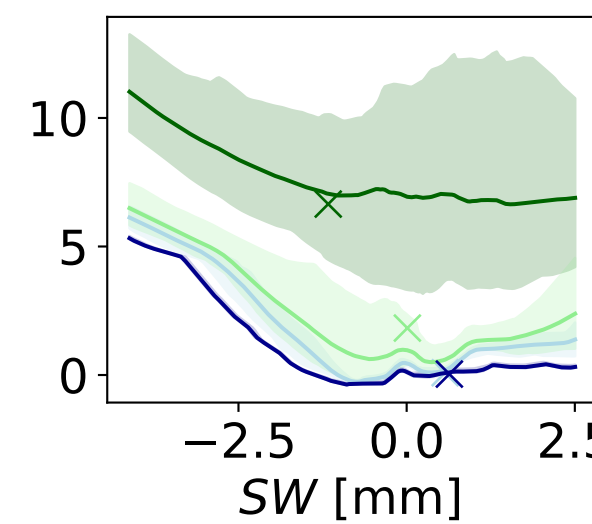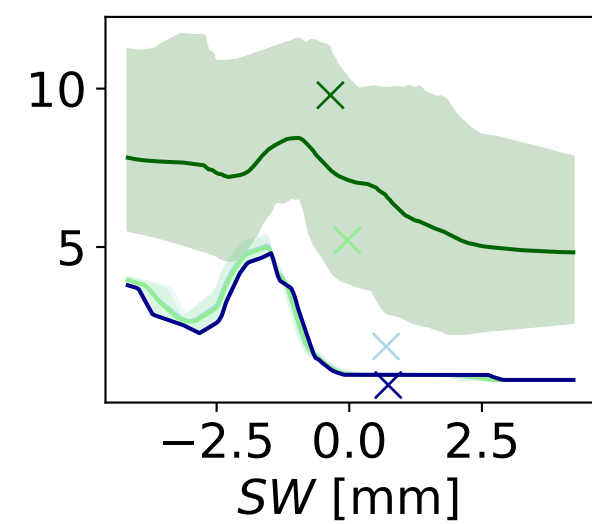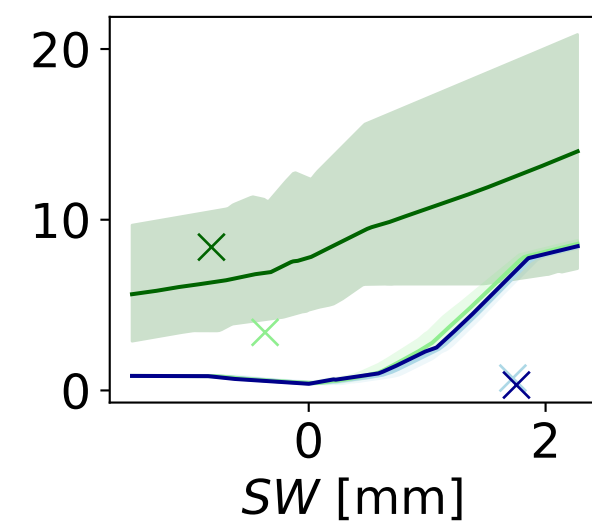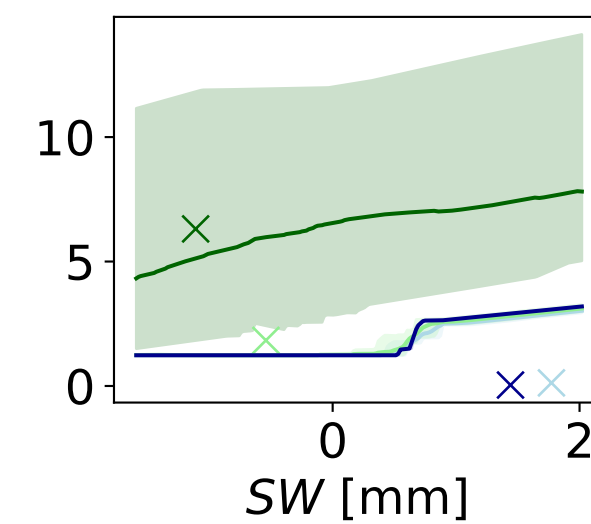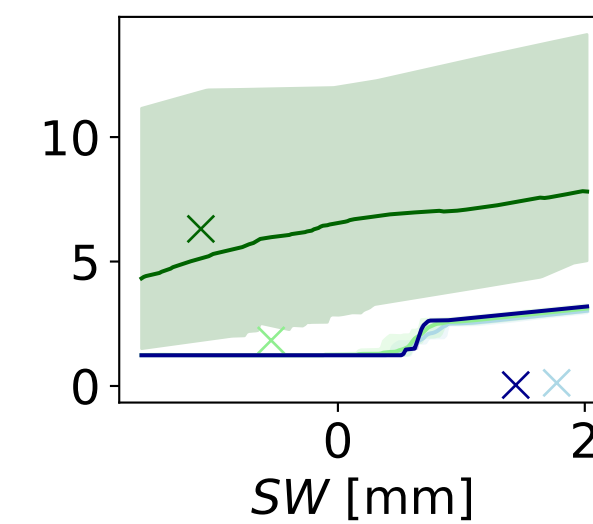

Spring Summer Autum Winter

Supplement: Supplementary file 1 — Data S1. [file ELE-27-0-s001.zip › via_res.pdf]
